# Glucocorticoid Resistance: Is It a Requisite for Increased Cytokine Production in Depression? A Systematic Review and Meta-Analysis

**DOI:** 10.3389/fpsyt.2019.00423

**Published:** 2019-06-28

**Authors:** Andrew J. Perrin, Mark A. Horowitz, Jacob Roelofs, Patricia A. Zunszain, Carmine M. Pariante

**Affiliations:** ^1^Stress, Psychiatry and Immunology Laboratory, Institute of Psychiatry, Psychology & Neuroscience, King’s College London, London, United Kingdom; ^2^Clinician Investigator Program and Department of Psychiatry, Faculty of Medicine, University of British Columbia, Vancouver, BC, Canada

**Keywords:** depression, cytokines, glucococorticoids, glucocorticoid resistance, inflammation

## Abstract

**Background:** Glucocorticoid resistance—reduced function of the glucocorticoid receptor (GR)—is seen in many depressed patients. It is argued that this resistance to glucocorticoids leads to failure of normal feedback regulation on the immune system. High levels of pro-inflammatory cytokines result.

**Purpose:** We sought to identify evidence supporting or refuting a link between glucocorticoid resistance and immune dysregulation in depression and to summarize retrieved evidence in aggregate form.

**Methods:** We systematically reviewed and meta-analyzed studies that examined cytokine levels in depressed patients compared with controls and that also reported a measure of glucocorticoid resistance. These measures included plasma cortisol, the dexamethasone suppression test (DST), *GR* expression levels, and the results of *in vitro* assays of GR function. We conducted four separate meta-analyses to test for moderating effects of glucocorticoid resistance on cytokine production in depression.

**Results:** After sub-grouping 32 studies by the ratio of cortisol levels in patients compared with controls, we observed a trend for increasing glucocorticoid resistance (i.e., the most hypercortisolemic patients) to be associated with increased production of interleukin (IL)-6 [*d* = 0.94; 95% CI (0.29, 1.59)] and tumour necrosis factor (TNF)-α [*d* = 0.46; 95% CI (0.12, 0.79)]. We stratified nine studies that reported DST results by relative glucocorticoid resistance between patients and controls, identifying a trend for higher glucocorticoid resistance in patients, compared with controls, to be associated with higher cytokine production in patients (170 patients and 187 controls). This was particularly evident when studies were sub-grouped by source of cytokine—plasma (*d* = 1.04; 95% CI, 0.57–1.50) versus *in vitro* (*d* = 0.24; 95% CI, −0.20 to 0.67). Stratifying the four studies (147 patients and 118 controls) that used *in vitro* assays of GR function or *GR* expression to quantify glucocorticoid resistance revealed variable contributions to cytokine production in patients compared with controls (overall effect size: *d* = 1.35; 95% CI 0.53–2.18). Combining our analyses of studies that reported DST results with those that used *in vitro* assays of GR function or *GR* expression to quantify glucocorticoid resistance (302 patients and 277 controls), we noted that although depressed patients produced more cytokines than controls (*d* = 1.02; 95% CI, 0.55–1.49), there was no evident positive correlation between glucocorticoid resistance and inflammation.

**Conclusions:** Our work provides some support for a model conceptualizing glucocorticoid resistance as a requisite for increased inflammation in depression. The limited number of studies identified highlights the need for purpose-designed investigations that directly examine the relationship between glucocorticoid resistance and cytokine production in depression.

## Introduction

Endogenous glucocorticoids play an essential role in driving adaptive responses to stress. They increase available blood glucose and initiate lipolysis for increased metabolic demands under stress, alter behavioral responses to stress, and modulate stress-induced immune function to prevent overactivation and consequent damage to host tissues (1, 2).

Secretion of endogenous glucocorticoids is tightly controlled by the hypothalamic–pituitary–adrenal axis (HPA axis). Corticotropin releasing factor (CRF) produced in the periventricular nucleus of the hypothalamus triggers release of adrenocorticotropin (ACTH) by the anterior pituitary. ACTH in turn triggers release of glucocorticoids, especially cortisol in humans, from the adrenal cortex (3). Importantly, secreted glucocorticoids engage feedback mechanisms in the anterior pituitary and the hypothalamus to limit further secretion of ACTH and CRF, respectively.

A large body of work has identified that in diseases of chronic stress, disruptions in the normal regulation of the HPA axis are present. In depressed patients, increased cortisol levels (4) that are resistant to feedback regulation by the HPA axis have been detected (5, 6). Similar increases in glucocorticoids and disruptions of HPA axis regulation in non-depressed patients are associated with the Cushing syndrome of glucocorticoid excess (7), yet depressed patients with elevated cortisol levels do not manifest the same syndrome. This observation argues for the presence of a resistance to high glucocorticoid levels in depressed patients. Multiple mechanisms have been invoked to explain this, including: impairments in glucocorticoid receptor (GR) function, changes in *GR* expression, alterations in glucocorticoid bioavailability through modified protein binding in the serum, changes in the HPA axis feedback rheostat, and impacts on the ability of the immune system to modulate glucocorticoid function (3). Indeed, proinflammatory cytokines can also feedback on the hypothalamus and anterior pituitary, for example, at times increasing HPA axis activity through modulation of GR function and expression (3, 8).

A reciprocal relationship between glucocorticoids and immune function also exists—high levels of glucocorticoids are known to strongly inhibit immune function. This well-known property of glucocorticoids is exploited in the clinical treatment of inflammatory and autoimmune diseases (2, 9) and is believed to play a role in protecting the nervous system from an over-active inflammatory response (10). In depression, high levels of glucocorticoids can co-exist with high levels of pro-inflammatory cytokines such as interleukin (IL)-1β, IL-6, and tumour necrosis factor (TNF)-α (8, 11). The concurrent presence of high glucocorticoid levels and high cytokine levels in depressed patients creates a complex interplay between the immune system and the HPA axis. For example, high levels of glucocorticoids would be expected to dampen immune function and cytokine elaboration, but they do not always. Models to explain this phenomenon suggest that high levels of glucocorticoids in depression cause resistance to glucocorticoid feedback on the HPA axis and that this developed glucocorticoid resistance allows the escape of pro-inflammatory signaling pathways from normal feedback inhibition (11) through the mechanisms discussed above.

Many studies have focused on characterizing either elevated glucocorticoids or cytokine-mediated inflammation in depression. Few works have focused on the relationship between glucocorticoid resistance and inflammation in depression, and the few that have produced conflicting results. We were interested in exploring this relationship further and hoping to resolve inconsistencies between the results of individual studies. Therefore, we conducted a systematic review and meta-analysis of all published studies that simultaneously reported the results of indices of glucocorticoid resistance and cytokine levels in depression. Such indices of glucocorticoid resistance included plasma cortisol (4), the dexamethasone suppression test (DST) (12), *GR* expression (11), and *in vitro* functional assays of the GR (13). Classically in depression, plasma cortisol is elevated, and dexamethasone is unable to restore this elevation to normality. *GR* expression is downregulated, and *in vitro* functional assays of the GR show resistance to exogenous glucocorticoid actions. Using this knowledge, we examined for links between glucocorticoid resistance and the elaboration of high levels of pro-inflammatory cytokines in depression and summarized these results to produce aggregate effects. We hoped to identify enough aggregate evidence to allow us to resolve the conflict present in the primary literature.

## Methods

We conducted our search and review using the methods outlined by Preferred Reporting Items for Systematic Reviews and Meta-analyses (PRISMA) (14).

### Search Strategy

We reasoned that evidence of glucocorticoid resistance may manifest by either of three outcomes in depressed patients—elevated plasma cortisol compared with control; greater proportional abnormalities on the DST/other endocrine suppression test(s) compared with control; or evidence of glucocorticoid resistance only *in vitro* or through reduced *GR* expression compared with control.

Endocrine suppression tests assess the ability of exogenously supplied glucocorticoids to suppress endogenous effects of cortisol and other glucocorticoids. The DST is the most commonly used of such assays. The DST identifies glucocorticoid resistance in subjects by examining the ability of exogenously supplied dexamethasone to suppress plasma cortisol levels *in vivo*. Subjects in whom serum cortisol levels do not reduce following dexamethasone administration are classified as “non-suppressors” and display *in vivo* evidence of glucocorticoid resistance (12).

Evidence of glucocorticoid resistance in blood or *ex vivo* cells is determined using a similar premise, namely, the ability of exogenous glucocorticoids to suppress *in vitro* proliferation or function of immune cells isolated from depressed patients and controls, or by reduced *GR* expression in depressed patients compared with controls.

To ensure that all three manifestations of glucocorticoid resistance were explored, we conducted three separate yet complementary literature searches to assess all eventualities.

#### i) Glucocorticoid Resistance as Assessed by Elevated Plasma Cortisol in Depressed Patients, Compared to Controls

MEDLINE, EMBASE, PsycInfo, and the Cochrane Database were searched for articles from origin until October 31, 2018, using the following search strategy: (exp Depression OR exp Depressive Disorder) AND (exp Adrenal Cortex Hormones OR exp Pituitary-Adrenal System) AND (exp Cytokines OR exp Inflammation Mediators OR exp Leukocytes OR exp Macrophages). Results were limited to studies in humans and reported in English. Inclusion criteria were as follows: studies that reported a measure of cortisol (preferably morning) in each adult (age > 17 years) depressed patient and control, as well as corresponding cytokine levels (measured either from blood or from *in vitro* studies of blood or immune cells) in both subject populations, and depression diagnosed in patients using a standardized clinical/diagnostic interview. Criteria leading to study exclusion included the following: presence of bipolar, psychotic or substance use disorders in patients or controls; acute infection in patients or controls within 2 weeks of the study; and obesity [body mass index (BMI) > 30] in patients or controls.

Anticipating that a low number of studies would be retrieved by our search, we did not exclude studies in which patients or controls suffered from chronic medical conditions (e.g., endocrine, inflammatory, autoimmune, oral-dental, or neurologic disease) or in which immune-modulating therapies, such as glucocorticoids or biologics, were used in patients or controls so long as only a minority of subjects (<10%) possessed either of these respective characteristics, or in which matched controls, including matching for chronic medical conditions and immune modulating therapies, were used. We also did not exclude studies in which depressed patients experienced co-morbid anxiety disorders, so long as the anxiety disorder was not the primary diagnosis at the time of study.

The search was executed by AP. Retrieved titles and abstracts were screened by AP and MH to assess conformity with inclusion and exclusion criteria. Disputes about the appropriateness for study inclusion were resolved by AP. Articles deemed suitable for inclusion were retrieved in full text and examined by AP. Reference lists of retrieved articles were also examined to identify additional relevant articles not identified in our database searches.

#### ii) Glucocorticoid Resistance Measured by DST or Other Endocrine Suppression Test in Patients and Controls

A similar search strategy was employed to that above. Identical databases for the same periods were queried using the following search terms: (exp Depression OR exp Depressive Disorder OR exp Affective Symptoms) AND (exp Hydrocortisone OR exp Glucocorticoids OR exp Adrenal Cortex Hormones OR exp Pituitary-Adrenal System) AND (exp Receptors, Steroid OR exp Dexamethasone OR suppression test.mp. OR glucocorticoid resistance.mp). AND (exp Inflammation OR exp Inflammation Mediators OR exp Cytokines OR exp Leukocytes OR exp Macrophages). Results were again limited to studies in humans and reported in English. Inclusion criteria were as follows: studies that reported a result from an endocrine suppression test in each adult (age > 17 years) depressed patient and control, as well as corresponding cytokine levels (measured either from blood or from *in vitro* studies of blood or immune cells) in both subject populations; and depression diagnosed in patients using a standardized clinical/diagnostic interview. Criteria leading to study exclusion were the same as those enumerated above. The search was executed, and articles screened and retrieved as described above.

#### iii) Glucocorticoid Resistance Measured Only *In Vitro* or by GR Expression

Identical databases for the same periods as above were queried using the following search terms: (exp Depression OR exp Depressive Disorder) AND (exp Adrenal Cortex Hormones OR exp Pituitary-adrenal System OR Steroid Receptor) AND (exp Cytokines OR exp Inflammatory Mediators OR exp Leukocytes OR exp Macrophages). Search results were limited as above. Inclusion criteria were: studies that reported a result from an *in vitro* assay of GR function, such as suppression of *in vitro* proliferation of immune cells by exogenous glucocorticoids or measurement of cytokine production as an assay for GR function in blood, or *GR* expression in each adult (age > 17) depressed patient and control, as well as corresponding cytokine levels (measured from plasma) in both subject populations; and depression diagnosed in patients using a standardized clinical/diagnostic interview. Criteria leading to study exclusion were the same as above. The search was executed, and articles screened and retrieved as described above.

### Data Extraction

Means and standard deviations for individual cytokine level from depressed patients and controls were extracted from studies when reported. When such values were not reported, we contacted study authors to obtain either raw data or the necessary values. When there were gaps in the data set of a given study (i.e., not all subjects had reported a measure of glucocorticoid resistance and a measure of cytokine level) and we were unable to obtain additional data from the study authors to bridge these gaps, we included these studies as they represented a minority in our analysis. Such inclusions are noted in the presented summary tables.

In some studies, values for plasma cortisol and cytokines were not normally distributed. We estimated mean and standard deviations from reported medians, data ranges, and sample sizes using the method of Wan (15). Non-Gaussian data has been found to have limited impact on the outcomes of meta-analysis (16), and given the small number of studies retrieved, we felt that excluding such data would materially bias our analysis.

When depressed subjects were divided into subtypes of the illness (i.e., with atypical features, with melancholic features, etc.), we combined all listed sub-types into one group of depressed patients and calculated means and standard deviations for these single groups. Where studies reported data on more than one patient group (e.g., patients with depression and another disease, as well as patients with depression only), we extracted data for patients with depression only, unless matched controls were used.

In the case of endocrine suppression test results, we extracted counts of suppressors and non-suppressors from reported studies or unpublished data provided by study authors. Although the plasma cortisol level used to define non-suppression in the DST varied from study to study, all cut-offs exceeded the generally accepted value of 1.8 µg/dL (17). If a study used a significantly higher cut-off value, we did not modify suppression and non-suppression counts as for such studies we did not possess the raw post-dexamethasone cortisol values that would have allowed us to make such modifications.

*In vitro* studies commonly reported outcomes for assays of GR function as percentage of basal effect. We extracted the difference of these percentages from 100% for further use in our study. *GR* expression levels from whole blood were reported as fold-change and extracted as such.

### Meta-Analysis

Meta-analyses were conducted using RevMan5 (18) and effect sizes are reported as Cohen’s *d*. Cohen’s *d* is calculated as follows:

$$\frac{{\bar{x}}_{\text{depressed}}-{\bar{x}}_{\text{control}}}{\sqrt{\frac{\sigma_{\text{depressed}}^{2}-\sigma_{\text{control}}^{2}}{2}}}$$

We derived effect sizes from means and standard deviations of cytokine levels from depressed patients and controls. Where reported, we preferentially used plasma values of cytokines in our analysis. When stimulated cytokine levels from *in vitro* assays were used, we selected the stimulant level at which maximal response was noted by the study authors.

Since we identified experimental variability during our systematic review, we presumed that there would be heterogeneity in our meta-analysis attributable to this variability and therefore conducted analysis using a random effects model.

*A priori*, we hypothesized that relative glucocorticoid resistance differences between patients and controls would also contribute to the heterogeneity observed between studies. Thus, we constructed two measures of relative glucocorticoid resistance that would allow us to undertake modifier analysis.

#### i) Glucocorticoid Resistance as Assessed by Relative Plasma Cortisol Levels Between Depressed Patients and Controls

We presumed that glucocorticoid resistance would manifest by high levels of plasma cortisol. We therefore used the ratio of average plasma cortisol in patients to average plasma cortisol in controls to assess relative glucocorticoid resistance between the two subject groups. Subsequent modifier analysis sub-grouped effect sizes from studies into those studies where patients were hypercortisolemic compared to controls (ratio patient:control > 1.2), patients had essentially similar plasma cortisol levels to controls (ratio, 0.8 < patient:control < 1.2; “eucortisolemia”), and where patients displayed lower plasma cortisol levels than controls (ratio patient:control < 0.8; “hypocortisolemia”). This modifier analysis allowed us to examine effect sizes in studies in which patients may have displayed more glucocorticoid resistance than in controls and to compare these with effect sizes from studies where there was a reduced relative difference in presumed glucocorticoid resistance between patients and controls.

Since we decided to use such an approach in our meta-analysis, we report pooled effect sizes only for those cytokines whose values were reported by five or more retrieved studies.

#### ii) Glucocorticoid Resistance Measured by DST or Other Endocrine Suppression Test in Patients and Controls, or in *In Vitro* Studies of GR Function in Blood or Cells from Patients and Controls or from GR Expression Levels in the Blood of Patients and Controls

To assess relative differences in glucocorticoid resistance between patients and controls when an endocrine suppression test was used, we developed a continuous measure of this comparison—the “glucocorticoid resistance index.”

$$\frac{\text{proportion of }{\text{suppressors}}_{\text{control}}-\text{proportion of }{\text{suppressors}}_{\text{patients}}}{\text{proportion of }{\text{suppressors}}_{\text{control}}}$$

This measure of relative difference in glucocorticoid resistance varies between −1 (all controls glucocorticoid resistant and none of patients) and 1 (all patients glucocorticoid resistant and none of controls). Such an approach avoids the mathematical difficulties inherent to comparing numbers of non-suppressors in patients and controls. We used the “glucocorticoid resistance index” as a variable to rank retrieved studies by the relative difference in glucocorticoid resistance between patients and controls (i.e., studies listed first in **Figures 4**, **5**, and **6** are those in which most, if not all, patients are glucocorticoid resistant and few, if any, controls are glucocorticoid resistant).

To analyze relative glucocorticoid resistance using *in vitro* measures of GR function, we modified the “glucocorticoid resistance index” as follows:

$$\frac{\left[1-\text{proportion of basal}{}_{\text{control}}]-[1-\text{proportion of basal}{}_{\text{patients}}\right]}{\left[1-\text{proportion of basal}{}_{\text{control}}\right]}$$

where “basal” is assay output in the absence of exogenous glucocorticoid. This measure varies in an identical manner to the classic “glucocorticoid resistance index.” When reported, we preferentially used these measures of GR function instead of *GR* expression.

To analyze relative glucocorticoid resistance using *GR* expression, we modified the “glucocorticoid resistance index” as follows:

$$\frac{\text{GR expression}{}_{\text{control}}-\text{GR expression}{}_{\text{patients}}}{\text{GR expression}{}_{\text{control}}}$$

This measure varies identically to those discussed above.

As we retrieved insufficient studies to conduct meta-analysis by individual cytokine in this arm of analysis, we selected from each study the cytokine reported (if more than one was reported) using the following prioritization criteria: 1) for studies reporting an endocrine suppression test result in patients and controls, cytokines for which most of the other studies also reported a value; barring this, cytokines for which a plasma level, rather than an *in vitro* level, was reported; 2) for studies reporting an *in vitro* measure of GR function or *GR* expression in patients and controls, cytokines for which most of other studies also reported a value from plasma; barring this, cytokines for which the maximum positive effect size was demonstrated in plasma.

### Assessment of Heterogeneity

Heterogeneity in pooling effects sizes was first assessed visually on forest plots. Standard assessments of heterogeneity calculated by RevMan (ခτ^2^ and *I* ^2^) were further used to assess the contribution of heterogeneity between studies to overall appropriateness in pooling effect sizes. τ^2^ and *I* ^2^ were used to examine the impact of moderator analysis on pooled effects sizes generated in sub-group analysis.

### Sensitivity Analysis and Reporting Bias

We conducted standard serial exclusion of studies to assess for individual study effect on the overall effect size reported. Funnel plots were generated in RevMan (18).

## Results

As three separate yet complementary approaches were utilized to examine our question, we report results for each approach serially.

### i) Glucocorticoid Resistance as Assessed by Relative Plasma Cortisol Levels Between Depressed Patients and Controls

**Figure 1:**
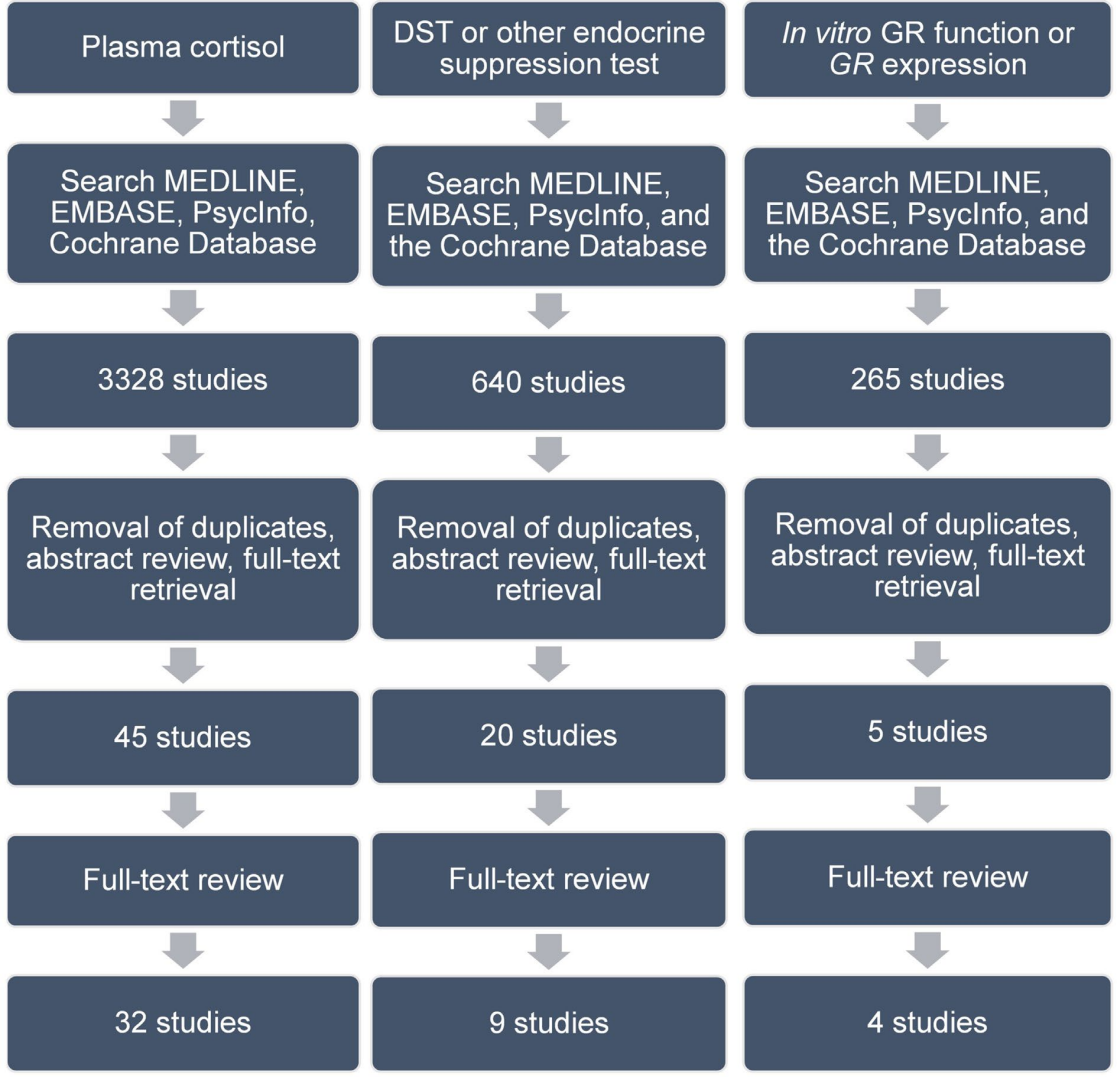
Search strategy and article review process. Details of this are found in text. Abbreviations are as follows: DST, dexamethasone suppression test; GR, glucocorticoid receptor.

There were 3,328 articles identified in our database search (**Figure 1**). After removal of duplicates and review of titles and abstracts to ensure that studies met our inclusion criteria, 45 articles were retrieved for full-text review. Twelve articles were excluded for the following reasons: 2 studies did not include a control group; 5 studies did not report a measure of cortisol; 1 study reported cytokine levels only after oral dexamethasone challenge of patients and controls; 1 study did not report cytokine measures; 1 study did not use a structured clinical/diagnostic interview to diagnose depression in patients or to exclude mental illness in controls; 2 studies included patients who suffered from bipolar, psychotic, or substance use disorders.

Further detailed review of the remaining 33 articles identified two articles that examined the same cytokine in the same patient population. We excluded one of these articles (19) to avoid duplication bias and included the other, which reported a more comprehensive analysis of the subjects (20). This left 32 articles to include in our review (**Table 1**).

**Table 1:** Studies included in analysis using relative plasma cortisol levels as a measure of glucocorticoid resistance.

Study	Patients	Control	Age	Medical co-morbidity	Psychiatric co-morbidity	Medications in patients	Anti-inflammatories	Diagnostic method	Cortisol source; sample timing/ detail	Cortisol level patients	Cortisol level control	Cortisol ratio—patients/control	Cytokine; source; sample timing/ detail	Stimulant	Cytokine level patient	Cytokine Level Control
Alesci (21)^a^	9 outpatients	9, matched	Adult	No	No	Yes	No	SCID-IV	Plasma; at 0800/after 30-min rest	9.7 +/− 1.0 (SEM) µg/dL	11.4 +/− 0.8 (SEM) µg/dL	0.85	IL-6; plasma; 0900/after 60-min rest	None	5.3 +/− 1.5 (SEM) pg/ml	3.4 +/− 0.6 (SEM) pg/ml
Allen (22)	37 combined inpatients and outpatients	20	Adult	No	No	Yes	No	SCID-IV	Salivary; 30-min post-awakening/variable waking time	12.24 +/− 3.55 (SD) nM	10.5 +/− 1.5 (SD) nM	1.16	IL-6, IL-8, IL-10, IFN-γ; plasma; between 0800 and 1100	None	IL-6—2.72 +/− 1.67 (SD) pg/ml; IL-8— 2.23 +/− 3.55 (SD); IL-10 – 7.27 +/− 2.73 (SD) pg/ml; IFN-γ—17.7 +/− 4.27 (SD) pg/ml	IL-6—2.8 +/− 0.3 (SEM) pg/ml; IL-8 - 11 +/− 1.0 (SEM) pg/ml; IL-10 - 6.5 +/− 1.0 (SEM) pg/ml; IFN-γ—15.2 +/− 2.3 (SEM) pg/ml
Anisman (23)	45 outpatients	27	Adult	No	No	No	No	Patient—clinical interview; control—MINI	Plasma; between 0730 and 0900/after 20-min rest	12.61 +/− 3.59 (SD) µg/dL	16.18 +/− 5.45 (SD) µg/dL	0.78	IL-1β, IL-2; *ex vivo* cells stimulated	PHA	IL-1β—1,281.1 +/− 36.19 (SD) µg/ml; IL- 2—512.89 +/− 22.9 (SD) pg/ml	IL-1β—1,400 +/− 519.62 (SD) µg/ml; IL-2—980 +/− 519.62 (SD) pg/ml
Bauer (24)	36 inpatients	31	Adult	No	No	Yes	No	Patient—clinical interview; control—clinical interview	Salivary; at 1000/prior to phlebotomy	11.88 +/− 3.5 (SD) nM	9.1 +/− 5.7 (SD) nM	1.31	IL-2, TNF-α; *ex vivo* cells stimulated	PHA (IL-2), LPS (TNF-α)	IL-2—338.5 +/− 69.8 (SEM) pg/ml; TNF-α—880 +/− 90 (SEM) pg/ml	IL-2 297.1 +/− 101.7 (SEM) pg/ml; TNF-α—890 +/− 90 (SEM) pg/ml
Carvalho (25)	19 inpatients	21	Adult	No	No	Yes	No	SCID-IV	Plasma; morning	340 +/− 30 (SEM) pg/ml	200 +/− 20 (SEM) pg/ml	1.7	IL-4, IL-6, IL-10, MCP-1, TNF-α, VEGF; plasma; morning	None	IL-4—2.6 +/− 0.1 (SEM) pg/ml; IL-6 - 3.0 +/− 0.1 (SEM) pg/ml; IL-10—1.7 +/− 0.05 (SEM) pg/ml; MCP-1—150 +/− 15 (SEM) pg/ml; TNF-α—2.9 +/− 0.1 (SEM) pg/ml; VEGF—14.5 +/− 1.5 (SEM) pg/ml	IL-4—3.2 +/− 0.5 (SEM) pg/ml; IL-6 - 1.9 +/− 0.15 (SEM) pg/ml; IL-10—1.3 +/− 0.05 (SEM) pg/ml; MCP-1 - 120 +/− 10 (SEM) pg/ml; TNF-α- 2.3 +/− 0.2 (SEM) pg/ml; VEGF - 23 +/− 2 (SEM) pg/ml
Carvalho (13)	15, inpatients	28	Adult	No	No	Yes	No	Patient—SCID–IV; control – not specified	Plasma; at 1000	429.4 +/− 55.4 (SEM) nM	242.2 +/− 14.8 (SEM) nM	1.77	IL-6; plasma and whole blood stimulated; at 1000 (plasma)	LPS (whole blood)	Plasma—3.0 +/− 0.29 (SEM) pg/ml; whole blood stimulated—1,025 +/− 175 (SEM) pg/ml	Plasma—2.4 +/− 0.1 (SEM) pg/ml; whole blood stimulated—875 +/− 150 (SEM) pg/ml
Cubala (20)^b^	20 outpatients	20	Adult	No	No	No	No	SCID-IV	Plasma; between 0800 and 0900/after 45-min rest	426.95 (369.2, 484.6) (95% CI) nM	322 (264.5, 379.5) (95% CI) nM	1.33	CRP; salivary; between 0800 and 0900/after 45-min rest	None	108.07 +/− 97.74 (SD) pg/ml	115.7 +/− 80.18 (SD) pg/ml
Darko (26)	20 inpatients	20	Adult	No	No	No	No	SCID-III	Plasma; between 0830 and 0930	20 +/− 5 (SD) µg/dL	16 +/− 5 (SD) µg/dL	1.25	IL-2; *ex vivo* cells stimulated	PHA	3.3 +/− 6.0 (SD) IU/ml	3.0 +/− 3.2 (SD) IU/ml
Du (27)	21 outpatients	27	Adult	No	No	Not specified	No	Clinical interview	Salivary; at 0800	8.6 +/− 2.4 (SEM) pg/µL	8.4 +/− 1.5 (SEM) pg/µL	1.02	ECP, EOTAXIN-2, IFN-γ, RANTES, TNF-α; plasma; at 0800	None	ECP—8.9 +/− 0.6 (SEM) µg/L; EOTAXIN-2 − 306.9 +/− 72.7 (SEM) pg/ml; IFN-γ—149.5 +/− 10.1 (SEM) pg/ml; RANTES—3368.0 +/− 129.6 (SEM) pg/ml; TNF-α—132.3 +/− 9.8 (SEM) pg/ml	ECP—12.5 +/− 1.9 (SEM) µg/L; EOTAXIN-2—383.6 +/− 84.0 (SEM) pg/ml; IFN-γ—143.8 +/− 6.7 (SEM) pg/ml; RANTES—3,410.8 +/− 113.9 (SEM) pg/ml; TNF-α—126.8 +/− 8.8 (SEM) pg/ml
Fitzgerald (28)	19	38	Adult	No	No	Yes	No	Patient—clinical interview; control—not specified	Plasma; between 0900 and 1100	325.5 +/− 26.4 (SEM) nM	294.6 +/− 28.3 (SEM) nM	1.1	IL-6; TNF-α; plasma; between 0900 and 1100	None	TNF-alpha—22.02 +/− 3.62 picogram/ml (mean +/− SEM); *n* = 19, IL-6—1.18 +/− 0.12 picogram/ml; n = 19	TNF-alpha—12.10 +/− 2.56 picogram/mL (mean +/− SEM); n = 38, IL-6—0.73 +/− 0.11 picogram/ml; *n* = 38
Humphreys (29)	9 outpatients	11	Adult	No	No	No	No	Patient—SCID-IV; control—not specified	Plasma; at 0800/after 30-min rest	20.1 +/− 3.7 (SEM) µg/dL	19.5 +/− 7.7 (SEM) µg/dL	1.03	IL-6; *ex vivo* cells unstimulated and stimulated	LPS	Unstimulated—3,541.2 +/− 726.8 (SEM) pg/ml; stimulated – 19,867.7 +/− 3,649.2 (SEM) pg/ml	Unstimulated—380.4 +/− 77.5 (SEM) pg/ml; stimulated—33,142.2 +/− 1,547.2 (SEM) pg/ml
Jozuka (30)	17 outpatients	10	Adult	No	No	No	No	Clinical interview	Plasma; between 0900 and 1000	7.1 +/− 4.5 (SD) µg/dL	12.3 +/− 3.8 (SD) µg/dL	0.58	IL-2; plasma; between 0900 and 1000	None	542 +/− 111 (SD) pg/ml	344 +/− 98 (SD) pg/ml
Kaestner (31)	37 inpatients	37	Adult	No	No	No	No	SCID-IV	Plasma; at 0800	203.51 +/− 14.46 (SD) ng/ml	180 +/− 80 (SD) ng/ml	1.13	IL-1β, IL-1RA; plasma; at 0800	None	IL-1β—37.3 +/− 6.19 (SD) pg/ml; IL-1RA—2,224.32 +/− 47.81 (SD) pg/ml	IL-1β—21 +/− 27 (SD) pg/ml; IL-1RA—1,600 +/− 750 (SD) pg/ml
Kahl (32)	34 outpatients	25	Adult	No	No	Yes	No	Patient—SCID-IV; controls—standardized psychiatric interview	Plasma; between 0700 and 0800	579.1 +/− 162.6 (SD)	423.4 +/− 150.1 (SD) nM	1.37	IL-6, TNF-α; plasma; between 0700 and 0800	None	IL-6—1.5 +/− 0.8 (SD) pg/ml; TNF-α—1.7 +/− 1.5 (SD) pg/ml	IL-6—1.7 +/− 1.4 (SD) pg/ml; TNF-α —0.7 +/− 0.5 (SD) pg/ml
Kahl (33)	27 inpatients	19	Adult	No	No	Yes	No	Patient—SCID-IV; control—standard psychiatric interview	Plasma; between 0700 and 0800	556.7+/− 150.5 (SD) nM	412.3 +/− 123.4 (SD) nM	1.35	IL-6, TNF-α; plasma; between 0700 and 0800	None	IL-6—1.9 +/− 2.2 (SD) pg/ml; TNF-α—1.9 +/− 1.8 (SD) pg/ml	IL-6—1.8 +/− 1.4 (SD) pg/ml; TNF-α —0.8 +/− 0.5 (SD) pg/ml
Kahl (34)	18	20	Adult	No	No	Yes	No	Patient—SCID-IV; control—standardized psychiatric interview	Plasma; between 0700 and 0800	661 +/− 384 (SD) nM	554 +/− 119 (SD) nM	1.19	IL-6, TNF-α; plasma; between 0700 and 0800	None	IL-6—1.45 +/− 1.8 (SD) pg/ml; TNF-α—3.90 +/− 0.9 (SD) pg/mL	IL-6—0.76 +/− 0.33 (SD) pg/ml; TNF-α—1.99 +/− 0.51 (SD) pg/mL
Karlovic (35)	55 inpatients	18	Adult	No	No	Yes	No	SCID-IV	Plasma; between 0800 and 0900/after 30-min rest	711.24 +/− 26.9 (SD) nM	560 +/− 65.2 (SD) nM	1.27	IL-6, TNF-α; plasma; between 0800 and 0900/after 30-min rest	None	IL-6—2.83 +/− 1.70 (SD) pg/ml; TNF-α—6.47 +/− 2.57 (SD) pg/ml	IL-6—1.75 +/− 1.1 (SD) pg/L; TNF-α - 5.40 +/− 1.5 (SD) pg/L
Landmann (36)	22 outpatients	22	Adult	No	No	Yes	No	Patient—clinical interview; control—not specified	Plasma; at 0800	505 +/− 27 (SEM) nM	465 +/− 35 (SD) nM	1.09	IFN-γ, TNF-α; plasma (IFN-γ); at 0800 (IFN-γ), *ex vivo* cells stimulated (TNF-α)	LPS	IFN-γ—30 +/− 8 (SEM) ng/L; TNF-α—1.42 +/− 0.4 (SEM) ng/L	IFN-γ—17 +/− 4 (SEM) ng/L; TNF-α—2.01 +/− 0.49 (SEM) ng/L
Lamers (37)	233 inpatients and outpatients	543	Adult	Yes—CAD (∼5%); DM (∼5%)	Yes—anxiety disorders	Yes	Yes (∼5%)	Composite Diagnostic International Interview	Salivary; awakening response (area under curve to ground) at awakening, 30-, 45- and 60-min post-awakening/variable waking time	19.38 +/− 4.41 (SD) nM	18.47 +/− 6.85 (SD) nM	1.05	CRP, IL-6, TNF-α; plasma; not specified	None	CRP—1.53 +/− 1.24 (SD) mg/L; IL-6—0.9 +/− 0.95 (SD) pg/ml; TNF-α – 0.91 +/− 0.96 (SD) pg/ml	CRP—1.12 +/− 3.23 (SD) mg/L; IL-6—0.73 +/− 2.58 (SD) pg/ml; TNF-α—0.84 +/− 1.90 (SD) pg/ml
Lisi (38)^b^	8	10	Adult	No	No	Yes	No	MINI	Salivary; at 0800	0.49 +/− 0.08 (SEM) µg/dL	0.43 +/− 0.08 (SEM) µg/dL	1.14	IL-1β, IL-6; mRNA from *ex vivo* cells stimulated	LPS	IL-1β—595.86 +/− 930.1 (SD) U; IL-6 —1,322.65 +/− 1,740.07 (SD) U	IL-1β—300.37 +/− 442.48 (SD) U; IL-6—612.63 +/− 912.97 (SD) U
Lopes (39)	22 outpatients	15	Adult	No	No	Yes	No	SCID-IV	Salivary; at 0800/always prior to venipuncture	7.8 +/− 1.0 (SEM) nM	12.5 +/− 0.5 (SEM) nM	0.624	IL-2, IL-4, IL-6, IL-10, IFN-γ, TNF-α; *ex vivo* cells stimulated	PHA	IL-2—512.14 +/− 109.12 (SEM) pg/ml; IL-4—346.37 +/− 87.48 (SEM) pg/ml; IL-6—3,931.82 +/− 880.15 (SEM) pg/ml; IL-10—1617.94 +/− 413.02 (SEM) pg/ml; IFN-γ—2,390.71 +/− 548.54; *n* = 22; TNF-α—2034.02 +/− 491.16 (SEM) pg/ml	IL-2—1060.90 +/− 189.40 (SEM) pg/ml; IL-4—2997.29 +/− 1,710.04 (SEM) pg/ml; IL-6—4,867.81 +/− 1,532.65 (SEM) pg/ml; IL-10—2,467 +/− 956.16 (SEM) pg/ml; IFN-γ—2,813.09 +/− 767.76; TNF-α— 2,063.64 +/− 593.13 (SEM) pg/ml
Maes (40)	19 inpatients	10	Adult	No	No	Yes	No	Patient—SCID-III; control—not specified	Plasma; at 0800	18.92 +/− 4.32 (SD) µg/dL	21.65 +/− 10.10 (SD) µg/dL	0.87	IL-1β; *ex vivo* cells stimulated	PHA	2,225 +/− 1,773 (SD) pg/ml	1115 +/− 1105 (SD) pg/ml
Maes (41)	48 inpatients	32	Adult	No	No	Yes	No	Patient—SCID-IV; control—structured interview	Plasma; at 0900/after 30-min rest	9.7 +/− 4.5 (SEM) µg/dL	9.3 +/− 3.7 (SEM) µg/dL	0.96	IL-6, sIL-2R; plasma; at 0845/after 15-min rest	None	IL-6—3.5 +/− 0.3 (SEM) pg/ml; sIL-2R—293 +/− 69 (SEM) U/ml	IL-6—1.5 +/− 0.3 (SEM) pg/ml; sIL-2R—236 +/− 100 (SEM) U/ml
Maes (42)	17 inpatients	8	Adult	No	No	Yes	No	Patient—SCID-III; controls—not specified	Plasma; at 0800	19.34 +/− 4.53 (SD) µg/dL	22.7 +/− 10.6 (SD) µg/dL	0.85	IL-6; *ex vivo* cells stimulated	PHA	45.3 +/− 6.93 (SD) ng/ml	26.6 +/− 13.7 (SD) ng/ml
Marques-Deak (43)^a^	45–46 outpatients	36–39, matched	Adult	No	No	No	No	Patient—SCID-IV; control—not specified	Plasma; at 0800	11.6 +/− 3.8 (SD) µg/dL	12.4 +/− 5.5 (SD) µg/dL	0.94	IFN-γ, IL-1β, IL-6; plasma; at 0800	None	IFN-γ—197.4 +/− 230.8 (SD) IU/ml; IL-1β—36.4 +/− 18.5 (SD) ng/ml; IL-6—132.4 +/− 83.2 (SD) ng/ml	IFN-γ—148.4 +/− 149.8 (SD) IU/ml; IL-1β—35.2 +/− 14.1 (SD) ng/ml; IL-6—129.3 +/− 61.6 (SD) ng/ml
Martinac (44)	49 inpatients	40	Adult	No	No	No	No	Patient—MINI; control—not specified	Plasma; at 0800/after 30-min rest	748.6 +/− 419.31 (SD) nM	476 +/− 116.88 (SD) nM	1.57	CRP, IL-6, TNF-α; plasma; at 0800/after 30-min rest	None	CRP—1.4 +/− 0.84 (SD) mg/L; IL-6 - 2 +/− 0.38 (SD) pg/ml; TNF-α—5.9 +/− 2.29 (SD) pg/ml	CRP—0.7 +/− 0.31 (SD) mg/L; IL-6—1.0 +/− 0.77 (SD) pg/ml; TNF-α – 5.0 +/− 2.31 (SD) pg/ml
Nikkheslat (45)^b^	19–20 outpatients	27–33, matched	Geriatric (∼68–70)	Yes—past MI (∼40%); HTN (∼75%); DM (∼20%); dyslipidemia (∼60%)	No	Yes (∼40%)	No	Clinical Interview Schedule-Revised	Plasma; before 1000	CRP—288.80 +/− 119.29 (SD) nM; IL-6—290.79 +/− 123.91 (SD) nM	CRP—341.67 +/− 104.58 (SD) nM; IL-6—369.22 +/− 117.16 (SD) nM	CRP—0.85; IL-6—0.79	CRP, IL-6; plasma; before 1000	None	CRP—4.99 +/− 4.57 (SD) mg/L; IL-6—2.38 +/− 1.90 (SD) pg/ml	CRP—3.34 +/− 4.29 (SD) mg/L; IL-6—2.21 +/− 2.49 (SD) pg/ml
Rudzki (46)	34 outpatients	29	Adult	No	No	Yes	No	Clinical interview	Plasma; between 0800 and 0900	174.76 +/− 12.08 (SEM) µg/ml	136.35 +/− 10.29 (SEM) µg/ml	1.28	IL-1β, IL-6, TNF-α; plasma; between 0800 and 0900	None	IL-1β—0.122 +/− 0.14 (SEM) pg/ml; IL-6—2.07 +/− 2.58 (SEM) pg/ml; TNF-α—1.09 +/− 0.4 (SEM) pg/ml	IL-1β—0.43 +/− 0.26 (SEM) pg/ml; IL-6—1.26 +/− 0.1 (SEM) pg/ml; TNF-α—1.7 +/− 0.13 (SEM) pg/ml
Simmons (47)^b^	26 outpatients	28	Adult	No	No	Yes	No	SCID-IV	Salivary; between 1900 and 2200	CRP—1.31 +/− 0.66 (SD) nM; IL-1RA—1.31 +/− 0.65 (SD) nM; IL-6—1.29 +/− 0.65 (SD) nM	CRP—1.21 +/− 0.52 (SD) nM; IL-1RA—1.20 +/− 0.51 (SD) nM; IL-6—1.20 +/− 0.56 (SD) nM	CRP - 1.085; IL-1RA - 1.091; IL-6 - 1.075	CRP, IL-1RA, IL-6; plasma; at 1200	None	CRP—3.17 +/− 2.91 (SD) mg/L; IL-1RA—0.35 +/− 0.20 (SD) ng/ml; IL-6—1.06 +/− 0.48 (SD) pg/ml	CRP—2.54 +/− 2.54 (SD) mg/L; IL-1RA—0.36 +/− 0.28 (SD) ng/ml; IL-6—0.72 +/− 0.36 (SD) pg/ml
Trzonkowski (48)^a^	10 inpatients	10, matched	Geriatric (∼50–90)	Yes, multiple	Yes—MNCD (∼50%)	No	Yes	SCID-IV	Plasma; between 0700 and 0800	355 +/− 35 (SD) nM	280 +/− 20 (SD) nM	1.27	IL-6, TNF-α; plasma; between 0700 and 0800	None	IL-6—650 +/− 140 (SD) fg/ml; TNF-α – 0.6 +/− 0.3 (SD) pg/ml	IL-6—230 +/− 20 (SD) fg/ml; TNF-α —0.3 +/− 0.05 (SD) pg/ml
Verduijn (49)^a^	1,083 outpatients	228	Adult	Yes, multiple	Yes—Substance use	Yes	Yes (∼5%)	Composite Diagnostic International Interview	Salivary; awakening response (Area Under Curve to Ground) at awakening, 30-, 45- and 60-min post-awakening/variable waking time	19.4 +/− 7.4 (SD) nM	18.2 +/− 7.0 (SD) nM	1.07	CRP, IL-6; plasma; around 0800	None	CRP—1.39 +/− 3.59 (SD) mg/L; IL-6—0.80 +/− 2.63 (SD) mg/L	CRP—1.14 +/− 3.09 (SD) mg/L; IL-6—0.71 +/− 2.47 (SD) mg/L
Weinstein (50)^b^	14 outpatients	14	Adult	No	No	Yes	No	SCID-IV	Plasma; between 1200 and 1600/after 30-min rest	IL-6/CRP—12.05 +/− 6.10 (SD) U; TNF-α—12.48 +/− 6.16 (SD) U	IL-6/CRP—11.71 +/− 5.38 (SD) U; TNF-α—12.05 +/− 5.41 (SD) U	IL-6/CRP—1.029; TNF-α—1.036	CRP, IL-6, TNF-α; plasma; between 1200 and 1600/after 30-min rest	None	CRP—1.35 +/− 1.18 (SD) U; IL-6 - 3.0 +/−3.33 (SD) U; TNF-α—2.48 +/− 1.31 (SD) U	CRP—2.01 +/− 2.15 (SD) U; IL-6 - 1.23 +/− 1.13 (SD) U; TNF-α—3.11 +/− 1.83 (SD) U

Where only a single standardized interview is listed, it was applied to both patient and control. ^a^cortisol and cytokine levels were not reported for every patient and control; analysis based on mean and standard deviation reported in paper. ^b^analysis based on raw data provided by study authors. Abbreviations are as follows: CAD, coronary artery disease; DM, diabetes mellitus; MI, myocardial infarction; HTN, hypertension; MNCD, major neurocognitive disorder; SCID-IV, Structured Clinical Interview for DSM-IV; SCID-III, Structured Clinical Interview for DSM-III; MINI, Mini-international Psychiatric Interview; SD, standard deviation; SEM, standard error of the mean; PHA, phytohemagglutinin; ECP, eosinophil cationic protein; EOTAXIN-2, eosinophil chemotactic protein-2; IL-1RA, IL-1 receptor antagonist; MCP-1, monocyte chemoattractant protein-1; RANTES, Regulated on Activation, Normal T cell Expressed and Secreted.

All studies were either of a case–control design or a non-randomized cohort design. For the latter type of study, we extracted data only from the baseline timepoint. This removed the impact of treatment interventions and effectively transformed the extracted data into a case–control design.

All studies reported either plasma or salivary cortisol levels in patients and controls. One study reported both plasma and salivary cortisol as well as *GR* expression (45). Thirty (94%) studies collected blood or saliva for cortisol analysis in the morning, generally between the hours of 0700 and 1100 (**Table 1**). Of the two remaining studies, one collected samples for cortisol analysis in the afternoon (50) and the other did so in the evening (47). Nine studies specifically mentioned a rest period of 15 to 45 min prior to the collection of samples used for quantification of cortisol (20–22, 23, 29, 35, 41, 44, 50). The remaining studies did not comment on this subject. Only two studies reported cortisol awakening responses in the form of area under the curve with respect to ground (AUC_g_) as their cortisol outcome measure (37, 49). All other studies reported average cross-sectional cortisol levels at the time specified in **Table 1**.

Twenty-four (75%) studies reported plasma, blood or salivary levels of cytokines measured by either ELISA or mRNA expression. The remaining studies reported only cytokine or mRNA levels from *in vitro* analysis of whole blood or immune cells. For the 24 studies that reported plasma, blood or salivary levels of cytokines, 23 either collected cytokine samples concomitantly with cortisol samples or within 1 h of the collection of cortisol samples. The remaining study (47) collected cytokine samples at 1200 and cortisol samples between 1900 and 2200.

Of note, 29 (91%) studies reported results from patients and controls with no psychiatric nor medical co-morbidities. Two studies were conducted exclusively in the elderly (age range, 50–90 years) who displayed a number of medical co-morbidities, such as coronary artery disease, diabetes, osteoarthritis, and major vascular neurocognitive disorder (45, 48). Patient and control groups in these studies were equally matched for medical and psychiatric co-morbidities, including neurocognitive disorders (48).

Antidepressant medications were used in patients in 69% of reviewed studies. Only three studies included patients who were using anti-inflammatory medications including glucocorticoids (doses not reported), but in two of these studies, less than 5% of patients used these medications (37, 49), and in the other study (48), anti-inflammatory medication use was negated by the use of matched controls. A small number of studies (13%) had gaps in the reported data that could not be rectified by attempted contact with study authors (21, 43, 48, 49).

Seven studies reported C-reactive protein (CRP) levels (20, 37, 44, 45, 47, 49, 50), one study reported eosinophil cationic protein (ECP) levels (27), one study reported eosinophil chemotactic protein-2 (EOTAXIN-2) levels (27), five studies reported interferon-γ (IFN-γ) levels (22, 27, 36, 39, 43), six studies reported IL-1β levels (23, 31, 38, 40, 43, 46), two studies reported IL-1 receptor antagonist (IL-1RA) levels (31, 47), five studies reported IL-2 levels (23, 24, 26, 30, 39), two studies reported IL-4 levels (25, 39), 23 studies reported IL-6 levels (13, 21, 22, 25, 28, 29, 32–34, 35, 37, 38, 41–49–50), one study reported IL-8 levels (22), three studies reported IL-10 levels (22, 25, 39), one study reported monocyte chemoattractant protein-1 (MCP-1) levels (25), one study reported regulated on activation, normal T cell expressed and secreted (RANTES) levels (27), one study reported soluble IL-2 Receptor (sIL-2R) levels (41), 15 studies reported TNF-α levels (24, 25, 27, 28, 32–36, 37, 39, 44, 46, 48, 50), and one study reported vascular endothelial growth factor (VEGF) levels (25).

As stated previously, we only meta-analyzed data for a specific cytokine if five or more studies reported values in patients and controls. We therefore meta-analyzed data for CRP, IFN-γ, IL-1β, IL-2, IL-6, TNF-α, comparing depressed patients with controls.

Irrespective of glucocorticoid resistance levels, patient levels of CRP (*d* = 0.23; 95% CI, −0.01 to 0.46), IFN-γ (*d* = 0.22; 95% CI, −0.02 to 0.47), IL-1β (*d* = 0.18; 95% CI, −0.24 to 0.61), and IL-2 (*d* = −0.12; 95% CI, −1.04 to 0.80) were all not significantly different from control. The number of studies included for these cytokines only just met the minimum number articulated above and in most cases the distribution of studies between “cortisolemic” states was uneven, leading to insufficient study number in each sub-group to conduct a formal analysis (data not shown).

**Figure 2:**
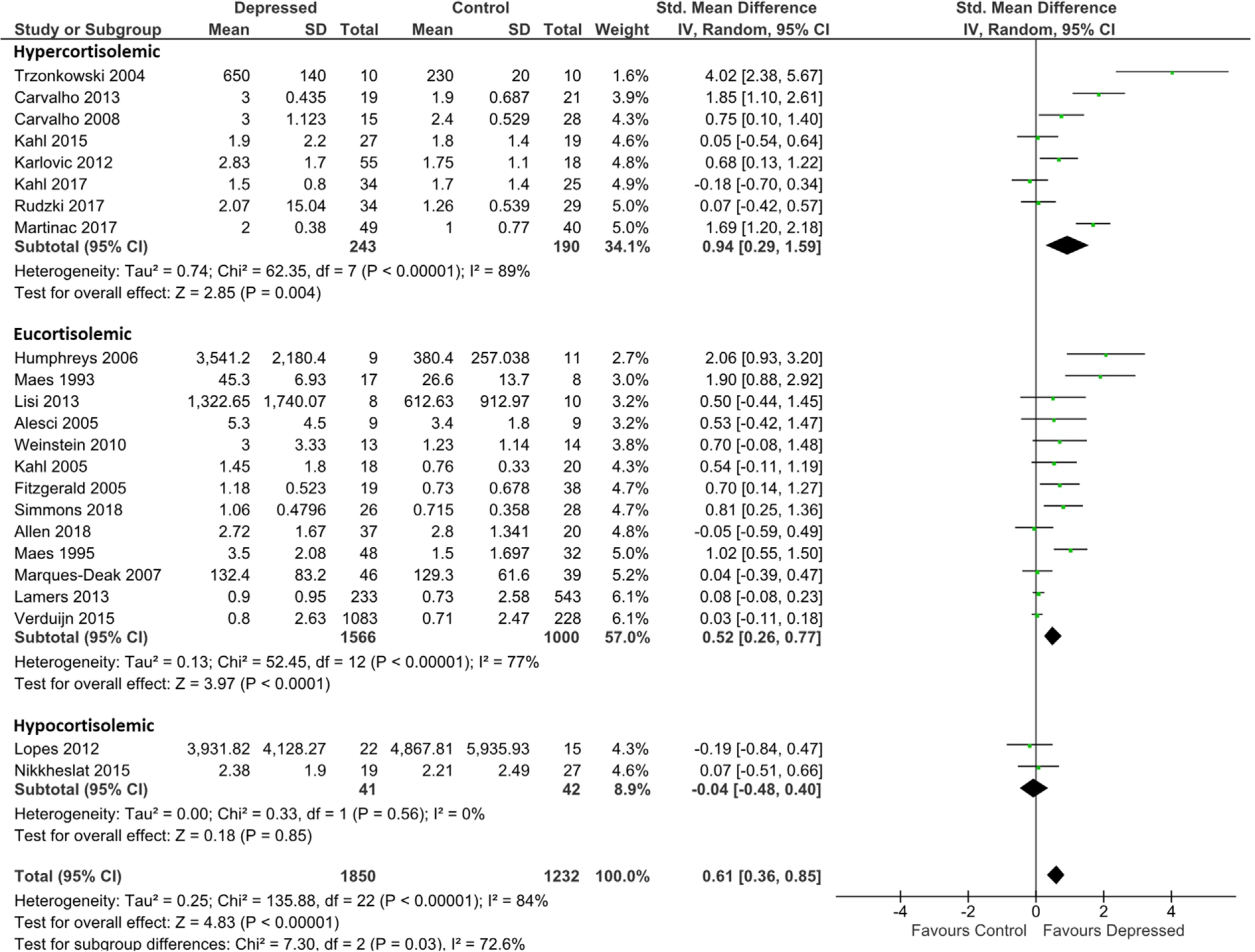
Forest plot analysing effect size for IL-6, sub-grouping studies by relative plasma cortisol levels. Hypercortisolemic corresponds to patient:control plasma cortisol ratio > 1.2; eucortisolemic corresponds to patient:control plasma cortisol ratio > 0.8 or < 1.2; hypocortisolemic corresponds to patient:control plasma cortisol ratio < 0.8.

IL-6 analysis was based on 1,850 patients and 1,232 controls. Overall effect size was 0.61 (95% CI, 0.36–0.85), demonstrating a significantly higher level of IL-6 in depressed patients than in controls (*p* < 0.0001; **Figure 2**). Heterogeneity was visually evident in this analysis, and this was reflected in statistical analysis of the same (τ^2^ = 0.25; *p* < 0.00001; *I* ^2^ = 84%). Overall effect size was insensitive to serial exclusion of studies.

Grouping studies by glucocorticoid resistance revealed that when glucocorticoid resistance in patients was higher in either the hypercortisolemic or eucortisolemic sub-groups (ratio patient:control > 1.2, or 0.8 < ratio patient:control < 1.2, respectively), overall effect size was significantly larger than in the two studies where patients were hypocortisolemic (ratio patient:control < 0.8) (**Figure 2**). Notably, hypercortisolemic patients (*d* = 0.94; 95% CI, 0.29–1.59) tended to produce more IL-6 compared with controls than did eucortisolemic patients (*d* = 0.52; 95% CI, 0.26–0.77), but this difference was not statistically significant (**Figure 2**). The difference in effect size observed between hypercortisolemic and eucortisolemic sub-groups was insensitive to serial exclusion of studies. Overall, sub-group difference testing revealed a significant result (χ^2^ = 7.3; *df* = 2; *p* = 0.03), but we did not interpret this to signify the existence of a true difference between the three sub-groups as the 95% confidence intervals associated with effect sizes in each sub-group sequentially overlapped as one moved from hypocortisolemic to eucortisolemic to hypercortisolemic.

Sub-grouping by presumed glucocorticoid resistance status did not appreciably reduce heterogeneity of the meta-analysis in the hypercortisolemic (ခτ^2^ = 0.74; *p* < 0.00001; *I* ^2^ = 89%) or the eucortisolemic (τ^2^ = 0.13; *p* < 0.00001; *I* ^2^ = 77%) sub-groups. The hypocortisolemic sub-group was homogeneous visually and statistically (**Figure 2**).

**Figure 3:**
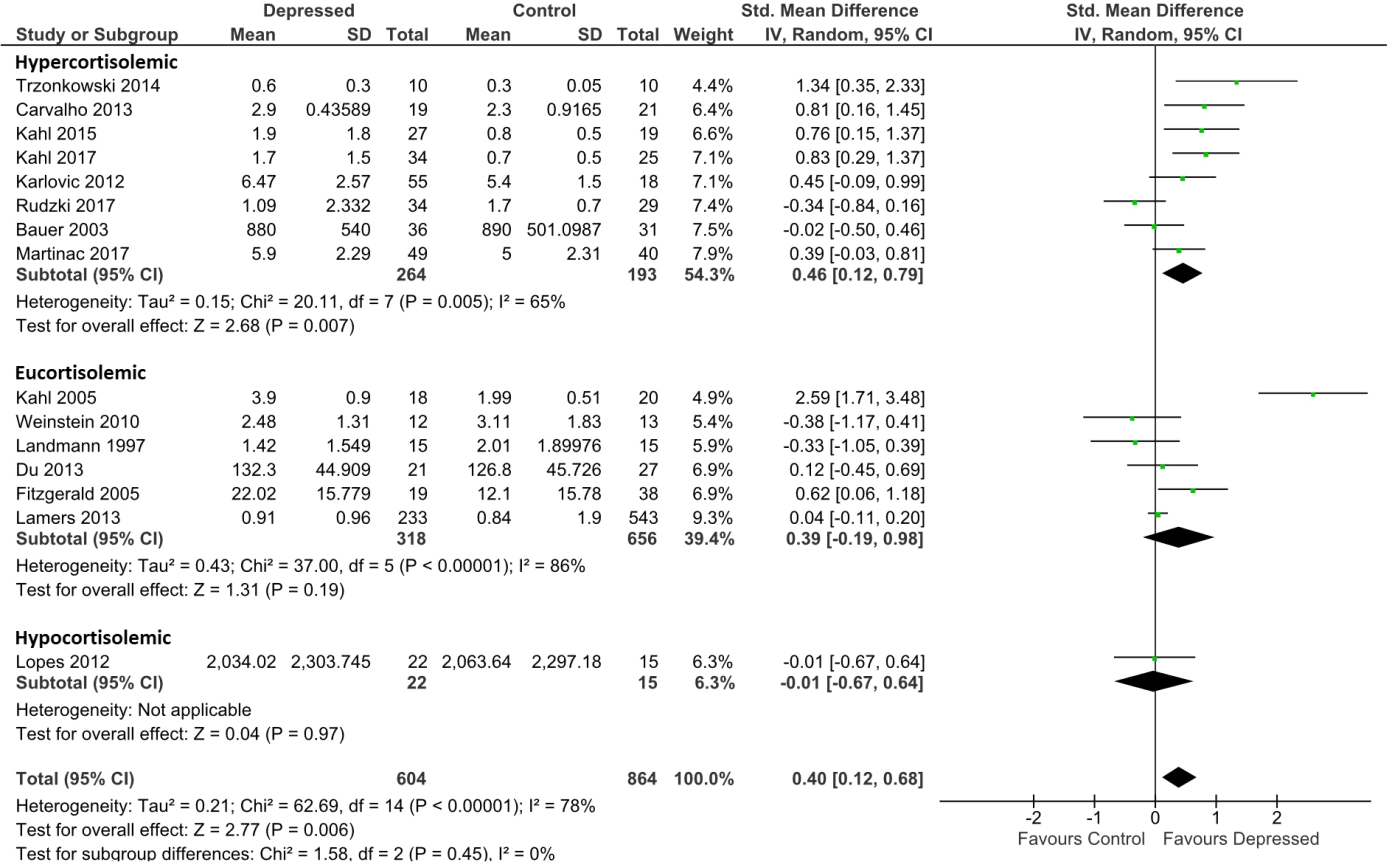
Forest plot analysing effect size for TNF-α, sub-grouping studies by relative plasma cortisol levels. Hypercortisolemic corresponds to patient:control plasma cortisol ratio > 1.2; eucortisolemic corresponds to patient:control plasma cortisol ratio > 0.8 or < 1,2; hypocortisolemic corresponds to patient:control plasma cortisol ratio < 0.8.

Analysis for TNF-α was based on data from 604 patients and 864 controls. Irrespective of presumed glucocorticoid resistance status, overall effect was moderate [*d* = 0.40; 95% CI, 0.12–0.68); *p* = 0.006]. Heterogeneity was visible on forest plots (**Figure 3**) and was reflected in measures of heterogeneity in the overall analysis (ခτ^2^ = 0.21; *p* < 0.00001; I^2^ = 78%). Subgrouping by glucocorticoid resistance status revealed a non-significant trend for hypercortisolemic patients to produce more TNF-α than eucortisolemic patients when compared with control (*d* = 0.46; 95% CI, 0.12–0.79 and *d* = 0.39; 95% CI, −0.19 to 0.98, respectively). An insufficient number of studies reported TNF-α levels in hypocortisolemic patients to allow comparison of this sub-group to the hypercortisolemic and eucortisolemic sub-groups (**Figure 3**). Significant heterogeneity was evident in both latter sub-groups (τ^2^ = 0.15; *p* < 0.005; *I* ^2^ = 65% and τ^2^ = 0.43; *p* < 0.00001; *I* ^2^ = 86%, respectively).

Censoring of Kahl et al. (34), which was a visual outlier in the eucortisolemic sub-group (**Figure 3**) accentuated the difference in effect size between the hypercortisolemic and eucortisolemic sub-groups (whole data set *d* = 0.46 and 0.39 versus adjusted *d* = 0.46 and 0.07, respectively), but this did not result in a statistically significant difference between the two sub-groups. Eucortisolemic sub-group heterogeneity significantly decreased with this censure (adjusted τ^2^ = 0.03; *p* = 0.18; *I* ^2^ = 36%). Serial exclusion of Rudzki et al. (46) and Trzonkowski et al. (48) from the hypercortisolemic sub-group did not significantly alter the difference in effect size between the hypercortisolemic and the eucortisolemic sub-groups. Only exclusion of Rudzki et al. (46) from the hypercortisolemic sub-group significantly reduced sub-group heterogeneity (adjusted τ^2^ = 0.06; *p* = 0.10; *I* ^2^ = 43%).

### ii) Glucocorticoid Resistance Measured by DST or Other Endocrine Suppression Test in Patients and Controls

Six hundred forty articles were identified in our database search (**Figure 1**). After removal of duplicates and review of titles and abstracts to ensure that studies met our inclusion criteria, 20 articles were retrieved for full-text review. 11 studies were excluded for the following reasons: four were conference abstracts for which we could not obtain associated study data; four studies did not include a control group; two studies did not use an independent measure of glucocorticoid resistance; one study did not measure glucocorticoid resistance. This left nine studies for our review (**Table 2**). All included studies were of a similar design to that described in the section Glucocorticoid Resistance as Assessed by Relative Plasma Cortisol Levels Between Depressed Patients and Controls.

**Table 2:** Studies included in analysis using endocrine suppression tests to measure relative glucocorticoid resistance.

Study	Patients	Controls	Medications used in patients?	Diagnostic method	Cortisol source	Endocrine suppression test used	Endocrine suppression test results	Glucocorticoid resistance index	Cytokine and source	Stimulant	Cytokine patient	Cytokine control
Bauer (24)	36 inpatients	31	Yes	Clinical interview	Salivary	DST	Patient—26/36; control—30/31 suppressors	0.25	IL-2; TNF-α; *ex vivo* cells stimulated	PHA (IL-2), LPS (TNF-α)	IL-2—338.5 +/− 69.8 (SEM) pg/ml; TNF-α—870 +/− 115 (SEM) pg/ml	IL-2—297.1 +/− 101.7 (SEM) pg/ml; TNF-α—880 +/− 100 (SEM) pg/ml
Carvalho (13)	15 inpatients	28	Yes	Patient—SCID-IV; control—not specified	Plasma	DST	Patient—0/15 suppressors; control—28/28 suppressors	1	IL-6; plasma and whole blood stimulated	LPS	Plasma—3.0 +/− 0.29 (SEM) pg/ml; whole blood stimulated—1,025 +/− 175 (SEM) pg/mL	Plasma—2.4 +/− 0.1 (SEM) pg/ml; whole blood stimulated - 875 +/− 150 (SEM) pg/ml
Fitzgerald (28)	19	38	Yes	Patient—clinical interview; control—not specified	Plasma	Skin blanching secondary to topical corticosteroid cream	Patient—0/19 suppressors; control—38/38 suppressors	1	IL-6, TNF-α; plasma	None	IL-6—1.18 +/− 0.12 (SEM) pg/ml; TNF-α—22.02 +/− 3.62 (SEM) pg/ml	IL-6—0.73 +/− 0.11 (SEM) pg/ml; TNF-α—12.10 +/− 2.56 (SEM) pg/ml
Humphreys (29)	9	11	No	Patient—SCID-IV; control—not specified	Plasma	DST	Patient—7/9 suppressors; control—10/11 suppressors	0.14	IL-6; *ex vivo* cells unstimulated and stimulated	LPS	Unstimulated—3,541.2 +/− 726.8 (SEM) pg/ml; stimulated—19,867.7 +/− 3649.2 (SEM) pg/ml	Unstimulated—380.4 +/− 77.5 (SEM) pg/ml; stimulated—33,142.2 +/− 1,547.2 (SEM) pg/ml
Landmann (36)	22 outpatients	22	Yes	Patient—clinical interview; control—not specified	Plasma	DST	Patient—21/22 suppressors; control—21/22 suppressors	0	IFN-γ, TNF-α; plasma (IFN-γ) and *ex vivo* cells stimulated (TNF-α)	LPS	IFN-γ—30 +/− 8 (SEM) ng/L; TNF-α—1.42 +/− 0.4 (SEM) ng/L	IFN-γ—17 +/− 4 (SEM) ng/L; TNF-α - 2.01 +/− 0.49 (SEM) ng/L
Lisi (38)	8	10	Yes	MINI	Salivary	DST	IL-1β—patient—5/8 suppressors, control—4/6 suppressors; IL-6—patient—6/8 suppressors, control—4/7 suppressors	IL-1β—0.0625; IL-6 —−0.31	IL-1β, IL-6; mRNA from *ex vivo* cells stimulated	LPS	IL-1β—595.86 +/− 930.1 (SD) U; IL-6 − 1322.65 +/− 1740.07 (SD) U	IL-1β—444.68 +/− 488.03 (SD) U; IL-6—695.3 +/− 1,027.38 (SD) U
Maes (40)	19 inpatients	10	Yes	Patient—SCID-III; control—not specified	Plasma	DST	Patient—13/19 suppressors; control—8/10 suppressors	0.145	IL-1β; *ex vivo* cells stimulated	PHA	2,225 +/− 1,773 (SD) pg/ml	1,115 +/− 1,105 (SD) pg/ml
Musselman (51)	11 inpatients and outpatients	9	No	SCID-III	Plasma	DST	Patient—8/11 suppressors; control—9/9 suppressors	0.27	IL-6; plasma	None	172.5 +/− 180.42 (SD) pg/ml	20.05 +/− 25.86 (SD) pg/ml
Soygur (52)	30 inpatients	30	Yes	SCID-IV	Plasma	DST	Patient—63% suppressors; control—100% suppressors	0.37	IL-6; plasma	None	17.75 +/− 5.15 (SD) ng/ml	9.5 +/− 4.66 (SD) ng/ml

Where only a single standardized interview is listed, it was applied to both patient and control. Abbreviations are as for **Table 1**, with the following exception: DST, dexamethasone suppression test.

Eight of nine studies evaluated glucocorticoid resistance using the DST. One study (28) used a previously validated cutaneous glucocorticoid resistance test (53) to evaluate glucocorticoid resistance. For this study, we used equivalent suppressor counts in patients and controls to calculate the “glucocorticoid resistance index.” Six studies reported unstimulated levels of cytokines, either measured by ELISA or by mRNA expression levels of cytokines (13, 28, 29, 36, 51, 52). Six studies used stimulation with mitogens to elicit increased cytokine secretion either from whole blood or *in vitro* culture of immune cells (13, 24, 29, 36, 38, 40), but only three studies relied exclusively on this method (24, 38, 40).

All studies were completed on adult patients and controls who had no medical nor psychiatric co-morbidities, including substance use. Seven of nine studies reported data from patients who were at least partially treated with antidepressants at the time of assay. No subjects in any of the studies were using anti-inflammatory medications or had a history or current manifestation of inflammatory illnesses.

One study reported IFN-γ levels (36), 2 studies reported IL-1β levels (38, 40), one study reported IL-2 levels (24), six studies reported IL-6 levels (13, 28, 29, 38, 51, 52), and three studies reported TNF-α levels (24, 28, 36). Five studies examined multiple cytokines (24, 28, 29, 36, 38), though not all studies reported a level for each cytokine examined (29).

**Figure 4:**
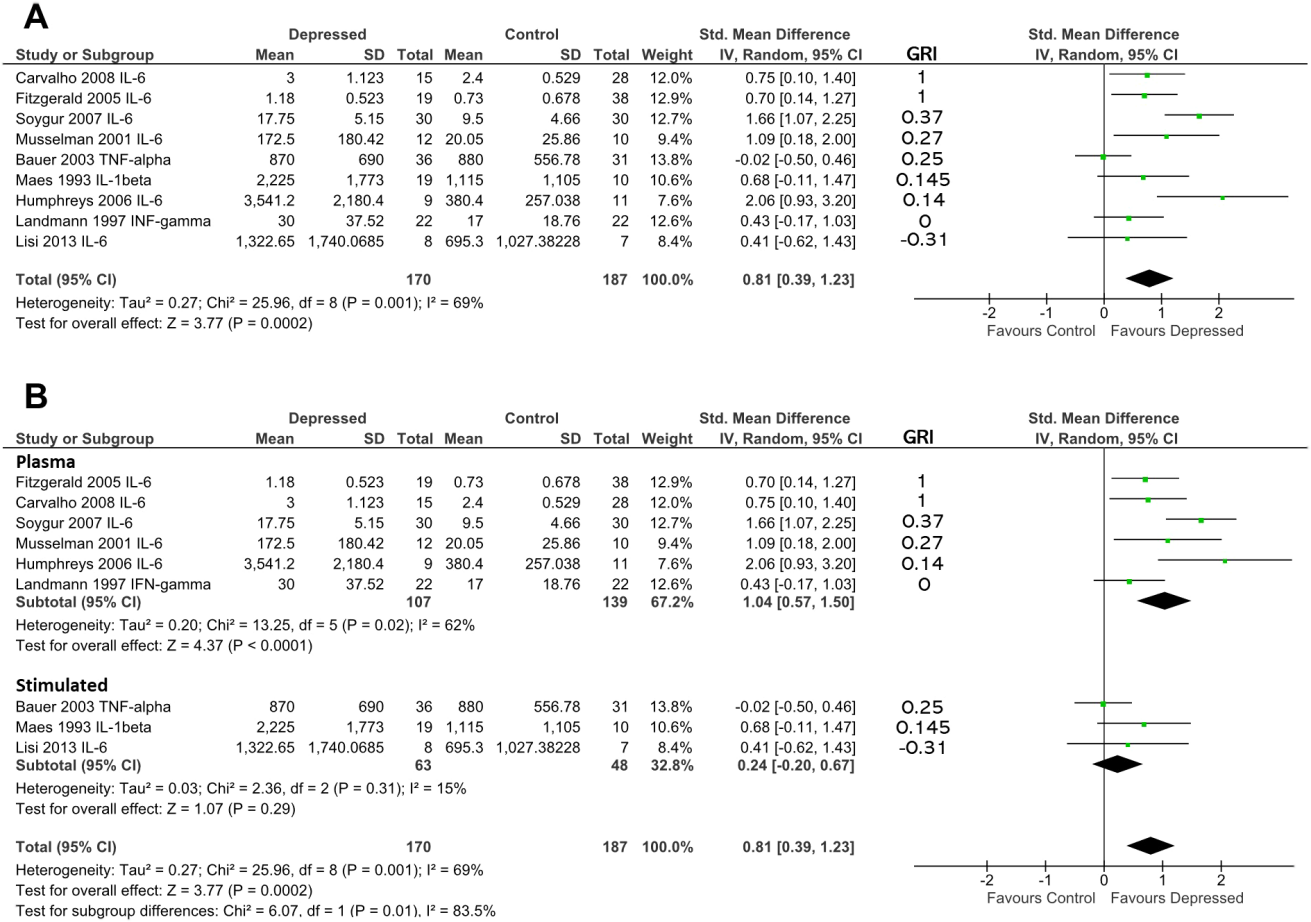
**(A)** Forest plot reporting effect size for cytokine stratified by “glucocorticoid resistance index” (GRI) calculated from DST or other endocrine suppression test. The specific cytokine selected for inclusion (based on criteria enumerated in Methods) is listed after the study name. Studies are ranked by GRI from high to low. **(B)** Forest plot of studies in **(A)** sub-grouped by source of cytokine. Stimulated levels were obtained from *in vitro* cultured cells using the stimulant specified in **Table 2**. Rank based on GRI (listed) as in **(A)**.

IL-6 levels from studies that examined this cytokine were used to determine effect sizes, whereas in the case of studies that did not examine IL-6, unstimulated levels of another cytokine were used (see **Figure 4**). This approach was taken to minimize heterogeneity introduced by pooling plasma and stimulated study results. All patients were at least as glucocorticoid resistant as controls.

When studies were ranked from high glucocorticoid resistance in patients to low glucocorticoid resistance using the “glucocorticoid resistance index,” there was a slight trend for studies that reported high glucocorticoid resistance in patients to produce larger effect sizes (based on 170 patients and 187 controls; **Figure 4A**). Examination of regression residuals did not support the existence of a significant trend, however (data not shown). Patients overall produced significantly higher levels of cytokines than did controls (*d* = 0.81; 95% CI, 0.39–1.23); *p* = 0.0002). Significant heterogeneity was noted in this analysis (ခτ^2^ = 0.27; *p* = 0.001; *I* ^2^ = 69%).

Since three of nine studies used in the above analysis only were able to contribute data obtained by stimulating *in vitro* cultured immune cells with mitogens, we hypothesized that this experimental dichotomy may have introduced heterogeneity that affected our overall analysis.

When we performed moderator analysis that separated studies into those where cytokine levels were obtained from plasma and those where cytokine values were obtained from *in vitro* stimulated immune cells, there was a reduction in heterogeneity within subgroups (plasma: τ^2^ = 0.20; *p* = 0.02; I^2^ = 62%; stimulated: τ^2^ = 0.03; *p* = 0.31; *I* ^2^ = 15%; **Figure 4B**). Furthermore, this analysis revealed the preservation of effect size in the plasma sub-group (*d* = 1.04; 95% CI, 0.57–1.50) and separation of this effect from that seen in the *in vitro* stimulated sub-group (*d* = 0.24; 95% CI, −0.20 to 0.67). The difference between these two sub-groups was statistically significant (χ^2^ = 6.07; *df* = 1; *p* = 0.01).

### iii) Glucocorticoid Resistance Measured in *In Vitro* or from *GR* Expression Levels in Patients and Controls

Two hundred sixty-five articles were identified in our database search (**Figure 1**). After removal of duplicates and review of titles and abstracts to ensure that studies met our inclusion criteria, five articles were retrieved for full-text review. One study was excluded since it assessed only depressive symptoms and did not assess for the presence of a major depressive episode using a standardized clinical/diagnostic interview. One study was excluded because it assessed only children aged 6 to 11 years. Through expert consultation during peer review, we identified one additional study that did not appear in our literature search. This left four studies for our review (**Table 3**). All studies were of a case–control design.

**Table 3:** Studies included in analysis using *in vitro* assays of GR function or *GR* expression to measure relative glucocorticoid resistance.

Study	Patients	Control	Age	Medical co-morbidity	Medications in patients	Diagnostic method	*GR* expression patients	*GR* expression control	*In vitro* assay % of basal with glucocorticoid patients	*In vitro* assay % of basal with glucocorticoid controls	Glucocorticoid resistance index	Cytokine measured	Cytokine level patient	Cytokine level control
Carvalho (13, 54)	15 inpatients	28	Adult	No	Yes	Patient—SCID-IV; control—not specified			77 +/− 7 (SEM) %	56 +/− 13 (SEM) %	0.25	IL-6; plasma	1025 +/− 175 (SEM) pg/ml	875 +/− 150 (SEM) pg/ml
Carvalho (55)^a^	47 inpatients	42	Adult	No	Yes	Patients—SCID-IV; control—clinical interview	0.8	1			0.2	IL-1β, IL-6, IL-8; plasma	IL-1β—58.75 +/− 43.93 (SD) pg/ml; IL-6—1.525 +/− 1.104 (SD) pg/ml; IL-8—55 +/− 40.55 (SD) pg/ml	IL-1β—22.5 +/− 13.79 (SD) pg/ml; IL-6 —0.2625 +/− 0.08 (SD) pg/ml; IL-8—22.5 +/− 18.39 (SD) pg/ml
Cattaneo (56)	74 outpatients	34	Adult	Not specifically excluded	Yes	Patient—Schedules for Clinical Assessment in Neuropsychiatry; control—Psychosis Screening Questionnaire	0.85 +/− 0.01 (SEM)	1.03 +/− 0.02 (SEM)			0.17	IL-1A, IL-1B, IL-4, IL-6, IL-7, IL-8, IL-10, MIF, TNF; whole blood	IL-1α—1.00 +/− 0.02 (SEM); IL-1β—1.51 +/− 0.03 (SEM); IL-4—0.90 +/− 0.02 (SEM); IL-6—1.32 +/− 0.01 (SEM); IL-7—0.99 +/− 0.02 (SEM); IL-8—1.01 +/− 0.01 (SEM); IL-10—1.02 +/− 0.01 (SEM); MIF—1.30 +/−0.03 (SEM); TNF—1.55 +/− 0.04	IL-1α—0.96 +/− 0.04 (SEM); IL-1β—1.03 +/− 0.03 (SEM); IL-4—0.99 +/− 0.02 (SEM); IL-6—1.08 +/− 0.02 (SEM); IL-7—1.03 +/− 0.05 (SEM); IL-8—1.00 +/− 0.04 (SEM); IL-10—1.00 +/− 0.02 (SEM); MIF—0.98 +/− 0.04 (SEM); TNF—0.97 +/− 0.04 (SEM)
Nikkheslat (45)^b^	11 outpatients	14, matched	Geriatric (∼68-70)	Yes – past MI (∼40%); HTN (∼75%); DM (∼20%); Dyslipidemia (∼60%)	Yes (∼40%)	Clinical Interview Schedule – Revised			63.05 +/− 20.57 (SD) %	63.63 +/− 15.13 (SD) %	-0.016	CRP, IL-6; plasma	CRP—6.24 +/− 4.03 (SD) mg/L; IL-6—1.82 +/− 1.43 (SD) pg/ml	CRP—3.79 +/− 4.83 (SD) mg/L; IL-6—2.55 +/− 2.49 (SD) pg/ml

Where only a single standardized interview is listed, it was applied to both patient and control. ^a^identified during peer review process. ^b^analysis based on raw data provided by study authors. Abbreviations are as for **Tables 1** and **2**, with the following exception: MIF, macrophage migration inhibitory factor.

Two of four studies used suppression of lipopolysaccharide (LPS)-induced IL-6 production by exogenous glucocorticoids to measure glucocorticoid resistance (13, 45, 54). Two studies used *GR* expression to assess glucocorticoid resistance (55, 56). Patients in all studies generally displayed at least as much glucocorticoid resistance as controls did (**Table 3**).

Three of four studies examined adult patients and controls with no medical or psychiatric co-morbidities. The remaining study (45) examined geriatric patients only (age range 68–70). This latter study was conducted in subjects already known to suffer from coronary artery disease and thus both patients and controls displayed several cardiac co-morbidities. These included hypertension (∼75% prevalence), dyslipidemia (∼60% prevalence), diabetes (∼20% prevalence), and previous myocardial infarction (∼40% prevalence), although the prevalence of these co-morbidities were relatively equal between patients and controls.

The prevalence of patient antidepressant usage in each study varied between ∼40% and 100%. None of the reviewed studies included patients or controls who were taking anti-inflammatory medications.

The most commonly reported cytokine level was IL-6, which was reported by all studies (13, 45, 54–56). IL-6 levels were used from three studies (13, 55, 56) to determine effect size, whereas CRP was used for the other study (45).

**Figure 5:**
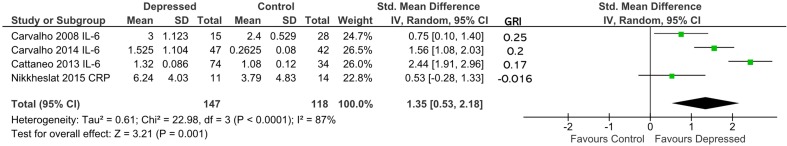
Forest plot reporting effect size for cytokine stratified by “glucocorticoid resistance index” (GRI) calculated from *in vitro* GR functional assay or GR expression. The specific cytokine selected for inclusion (based on criteria enumerated in Methods) is listed after the study name. Studies are ranked by GRI from high to low.

Overall effect size, based on 147 patients and 118 controls, was significantly different from 0 (*d* = 1.35; 95% CI, 0.53–2.18; *p* = 0.001; **Figure 5**). Heterogeneity was high (ခτ^2^ = 0.61; *p* < 0.0001; *I* ^2^ = 87%). Sensitivity analysis demonstrated that the overall effect size was mildly sensitive to exclusion only of Carvalho et al. (55) (adjusted effect size following study removal of *d* = 1.26; 95% CI, −0.01 to 2.53). Ranking studies by calculated glucocorticoid resistance did not reveal an obvious positive association between glucocorticoid resistance and cytokine production (**Figure 5**).

**Figure 6:**
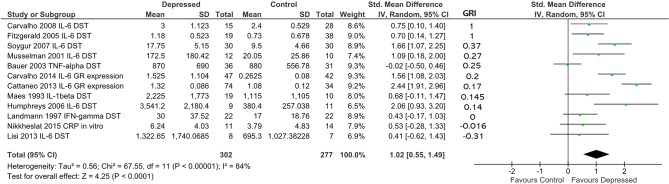
Forest plot reporting effect size for cytokine stratified by “glucocorticoid resistance index” (GRI) calculated from DST or other endocrine suppression test results, *in vitro* GR functional assay or *GR* expression. Studies are ranked by GRI from high to low. All study data included in this figure were previously shown in **Figures 4** or **5**. Only the data for Carvalho et al. (13) from **Figure 4A** was used in this analysis as inclusion of data from this study from both **Figure 4A** and **Figure 5** would have introduced duplication bias. Listed beside each study is the glucocorticoid resistance outcome measure that was used to calculate the GRI.

Building on our ability to standardize measurements of glucocorticoid resistance obtained through DST or other endocrine suppression tests, *in vitro* assays of GR function and *GR* expression levels using our glucocorticoid resistance index, we conducted a combined analysis of the studies included in **Figure 4** and **5**, using the effect sizes and glucocorticoid resistance index values reported in those figures (**Figure 6**). The data presented for Carvalho et al. (13) in **Figure 4A** was used for this analysis as inclusion of the data for this study from **Figure 5** as well would have resulted in duplication bias. For 302 patients and 277 controls, overall effect size was moderate (*d* = 1.02; 95% CI, 0.55–1.49; *p* < 0.0001], but heterogeneity was high (ခτ^2^ = 0.56; *p* < 0.00001; *I* ^2^ = 84%). Ranking studies from high to low glucocorticoid resistance index did not reveal a significant trend for higher inflammation to be associated with higher levels of glucocorticoid resistance in patients compared with controls (**Figure 6**). This was confirmed by observation of regression residuals (data not shown).

## Discussion

Individual studies have reported a possible positive association between glucocorticoid resistance and cytokine-mediated inflammation in depression. Other studies have failed to find evidence of the same phenomenon. The conflicting nature of the primary literature, combined with a consensus in the field that such a positive association indeed exists (11, 57), lead us to conduct the systematic review and meta-analytic work described above.

Initially we hypothesized that we would validate a positive association between glucocorticoid resistance and cytokine-mediated inflammation. Despite multiple methods of examining our hypothesis, however, we found only modest evidence to support this idea, with the largest effects noticeable when glucocorticoid resistance is measured by plasma or salivary cortisol. Furthermore, when we combined data in which glucocorticoid resistance was measured by the DST or other endocrine suppression tests, *in vitro* assays of GR function and *GR* expression, we were unable to increase the resolution of our analysis (**Figure 6**). The cross-sectional nature of our analysis may have obscured a significant mild-to-moderate trend in the relationship between inflammation and more systemic measures of glucocorticoid resistance (e.g., cortisol levels, DST) that would have otherwise been detected by prospective studies specifically designed to address this question. A limitation of the current analysis is the design of the included studies, but in the absence of further published studies, we feel that our work is unlikely to have missed a significant trend.

We took an unbiased and inclusive approach to our literature search and review methodology. Although this may have contributed to the increased heterogeneity of meta-analytic results observed, it also allowed as many possible manifestations of glucocorticoid resistance to be included. At the same time, each measure of glucocorticoid resistance that we invoked had its limitations. Serum cortisol levels are not always elevated in depressed patients, as demonstrated by our review [e.g., Refs. (39) and (45)] and by other studies that were outside of our inclusion criteria (58–60). Work in the elderly suggests that frailty drives exhaustion of the HPA axis in depression, possibly leading to hypocortisolemia (59, 61, 62) whereas chronic over-stimulation of the HPA axis in other age groups can cause long-term hypocortisolemia (61, 63). Furthermore, diurnal variations in cortisol levels are a well-known phenomenon of the HPA axis, with the highest cortisol levels evident in healthy subjects within the first hour of awakening from sleep. Most studies incorporated in our analysis (94%) measured cortisol in the morning hours, but not all targeted the hour following arousal (see **Table 1**). In fact, the hours of cortisol measurement spanned 0700–1100. This could have created variability in our assessment of glucocorticoid resistance in a given study, perhaps hampering the aggregation of data reported in **Figures 2** and **3**. On the other hand, the fact that most studies in our analysis restricted their measurement of cortisol to morning hours suggests that any variability that may have been introduced by this factor is likely minimal. Along the same line, levels of cytokines are also known to cycle in a circadian fashion. For example, the nadir of IL-6 in healthy subjects occurs between the hours of 0800 and 1000 (64), very near to the hours that cortisol experiences its zenith. In the analysis represented in **Figures 2** and **3**, almost all studies (96%) that reported plasma levels of cytokines collected samples concurrently with those used to assess cortisol levels. The adherence of most studies to rigid timing when measuring cortisol and cytokines means that our assessment of the impact of glucocorticoid resistance on inflammation using reported cortisol levels is unlikely to have been significantly affected by circadian variabilities in these two factors.

Inflammation associated with chronic medical conditions can also compound variabilities between the included studies (65). We invoked the DST as a measure of glucocorticoid resistance to circumvent some of these limitations, but by its nature the DST measures peripheral glucocorticoid resistance only. Challenges persist in translating findings from the DST into inferences about effects in the central nervous system (CNS) and DST results are subject to a number of confounders such co-morbid medical illnesses (66). Finally, *in vitro* measurements of glucocorticoid resistance represent the most controlled mechanism through which to test peripheral resistance to glucocorticoids but suffer, like the DST, from limitations in translating their findings to the CNS. *GR* mRNA expression levels suffer from the same limitation (17).

Combining all three of these measures of glucocorticoid resistance has allowed us to take a complementary approach in our analysis, maximizing the likelihood that any trends present would be detected. We found that studies that used *in vitro* measures of GR function and/or *GR* expression to measure glucocorticoid resistance delivered the largest aggregate effect size [*d* = 1.35; 95% CI (0.53, 2.18)], but this must be balanced against the observation that we did not detect an obvious positive association between glucocorticoid resistance and cytokine production in that analysis. Thus, it is tempting to speculate that *in vitro* measurement of GR function and/or *GR* expression may be more likely to detect a significant effect, perhaps by removing potential confounders from the analysis. Verification of this contention will require further studies using these measures to be conducted. The other two approaches used to assess glucocorticoid resistance in our study only delivered effect sizes between 0.5 and 1. These measures of glucocorticoid resistance may be subject to greater confounding. In the case of relative cortisol levels as a measurement of glucocorticoid resistance, studies included in our analysis obtained cortisol from varying sources (e.g., plasma and salivary) and the timing of cortisol measurement may have varied slightly between studies, even when a standardized time of collection was reported. Both possibilities could have created small variations between studies. In the case of the DST or other endocrine suppression tests as a measure of glucocorticoid resistance, a significant source of variation could be the different cut-off levels used by the included studies to classify a participant as a non-suppressor. As we mentioned in our Methods, all cut-offs used exceeded the generally accepted value of 1.8 µg/dL (17), but we were unable to create a standardized cut-off level as we did not possess the raw post-dexamethasone cortisol values that would have allow us to make such a determination. Taken together, we contend that although all measurements of glucocorticoid resistance are subject to confounding, *in vitro* measurements of GR function and/or *GR* expression may represent the method least likely to be subject to bias as *in vitro* assays allow tighter control of potential confounders and *GR* expression may represent a more durable measure of the effects of excess glucocorticoids in depressed patients than do cross-sectional measurements of cortisol or cortisol responses.

In this regard, using *GR* expression to identify glucocorticoid resistance in depression is a relatively new technique. Matsubara et al. (67) were among the first to identify decreased expression of the α transcript of the GR in depressed patients (*GRα* mRNA encodes the active form of the GR). Prior to this it was believed that the alterations in GR function in depression were driven primarily by post-translational modifications to the GR or its signalling pathways (68). Two subsequent studies (55, 56), including one from our group (56), confirmed that *GR* expression is indeed reduced in depressed patients. Carvalho et al. (55) found that increased levels of IL-8 in depressed patients correlated inversely with *GR* expression levels, suggesting that a certain level of intrinsic inflammation may occur independently of glucocorticoid resistance in depression, and that this inflammation plays a key role in the subsequent development of glucocorticoid resistance and further immune dysregulation through downregulation of *GR* transcript expression. This compounds the many impairments in GR function that occur during glucocorticoid resistance, including impaired translocation of the active GR from the cytoplasm to the nucleus, reduced affinity of the GR for its transcriptional binding sites and activation of competitor isoforms of the GR that impair the function of GRα (3). Although full exploration of the mechanism that underlies decreased *GR* expression in depression due to glucocorticoid resistance is still to come, the importance of *GR* expression as an independent signifier of glucocorticoid resistance is reinforced by the observation that *GR* expression levels did not correlate with the results of the DST in the population of depressed patients studied by Matsubara et al. (67). Furthermore, a reversal of decreased *GR* expression is seen when patients are treated with antidepressants (56), arguing that *GR* expression represents an important outcome of glucocorticoid resistance. Including *GR* expression analysis along with the DST and cortisol studies in any characterization of glucocorticoid resistance in depression is therefore highly desirable.

Our development of the glucocorticoid resistance index, a normalized way to compare glucocorticoid resistance between patients and controls across multiple studies, is rooted in the concept of relative differences. Thus, small absolute differences in resistance between patients and controls in a given study could result in a large relative difference. We feel that this limitation is acceptable as it is challenging to compare studies that report absolute counts of suppressors and non-suppressors in the DST or other *in vitro* measures of glucocorticoid resistance. Our index allows rapid conversion of absolute measures of glucocorticoid resistance into meaningful differences through which studies can be compared. As well, our index allows the conversion of count data on glucocorticoid resistance into a continuous measure of the same, possibly facilitating the use of meta-regression techniques in moderator analysis. In this study, we considered the use of meta-regression on our moderator, but preliminary analysis demonstrated no significant trends in plots of regression residuals (see Results). Nonetheless, we were able to capitalize on the power of the glucocorticoid resistance index when we combined data from studies using the DST or endocrine suppression tests, *in vitro* assays of GR function and *GR* expression in **Figure 6**.

Ideally, a common measure of glucocorticoid resistance would be used. The field as a whole has faced challenges in this task as it is unclear which measure should be adopted as standard (17). This likely relates in part to the variable results that are obtained when multiple measurements of resistance are applied to a single population of patients and controls. For example, when we used the cortisol levels of patients and controls, as well as *in vitro* measures of GR function to assess glucocorticoid resistance in the subjects analyzed by Nikkheslat et al. (45), we noted variances in the degree of glucocorticoid resistance inferred. We do not feel that this represents a systemic flaw in the data reported; rather, it is likely the result of the variability found when glucocorticoid resistance is quantified by different measures. We propose that multiple methods for evaluating glucocorticoid resistance are likely ideal for comparing data across many studies. Studies, such as those conducted by Miller et al. (69), which although not included in our meta-analysis due to conflict with our inclusion criteria, represent an ideal approach, assessing glucocorticoid resistance and inflammation through multiple independent measures.

Several studies that measured glucocorticoid resistance and cytokine production were identified by our review but were not be included in our meta-analyses. Doolin et al. (70) examined the relationship between waking salivary cortisol levels and mRNA expression of *IL-1β* and *IFN-γ* in whole blood. Measurements of both cytokine mRNA levels were non-Gaussian, with outcomes reported only as the results of non-parametric tests. We were unable to obtain raw data to circumvent this limitation. Nonetheless, Doolin et al. (70) report an inverse association between decreased morning cortisol reactivity, a marker of glucocorticoid resistance in depression, and *IL-1β* expression. No such relationship between cortisol reactivity and *IFN-γ* expression was found. Therefore, some support for an association between glucocorticoid resistance increased cytokine production in depression was found.

Stelzhammer et al. (71) used multiplex immunoassay and mass spectrometry to examine the relationship between plasma cortisol and IL-1RA, IL-16, and MIF in depressed and controls. They report higher levels of plasma cortisol and all three inflammatory markers in depressed patients but did not report parametric outcomes of statistical tests. Therefore, we were unable to incorporate this data into our analysis, but observe that elevated plasma cortisol and cytokine production co-existed in depressed patients (71), consistent with models promulgated in the literature (11).

Finally, Maes et al. (72) utilized the same patients as those in Maes et al. (40), undertaking *in vitro* analysis of both glucocorticoid resistance and cytokine production in cell culture supernatants. A strong association between elevated resistance to glucocorticoids (assessed using *in vitro* proliferation in the presence of exogenous glucocorticoids) and *in vitro* IL-1β production was noted. We elected to incorporate in our meta-analysis only studies that reported an *in vitro* measure of glucocorticoid resistance *and* plasma levels of cytokines in **Figure 5** and therefore, Maes et al. (72) was excluded from our analysis. Nonetheless, this study further supports the idea that elevated glucocorticoid resistance in depression is associated with increased cytokine-based inflammation.

Initially we considered more stringency in our exclusion criteria, such as removal of all studies in which medical co-morbidities were present in patients and controls, but we later re-considered this decision when we uncovered a body of literature that demonstrated changes in glucocorticoid resistance in patients who were both depressed and afflicted with a chronic illnesses such as cancer (51, 52). We amended our approach to include study participants who may have suffered from medical conditions, so long as their inclusion was balanced by matching controls. We reasoned that this would increase the resolution of our work, and we believe that it did without materially biasing our results, as censoring of the four studies examining glucocorticoid resistance and cytokine production in depressed patients with a variety of medical illnesses [e.g., asthma (27), cancers (51, 52), and cardiac disease (45)] did not significantly change our conclusions (data not shown). Unfortunately, the heterogeneity of these four studies in terms of the medical illnesses displayed by study subjects and the lack of similar published works precluded a more detailed analysis of this facet of inflammation in depression. These findings highlight the complex interplay between affective illness and medical illness that was discussed earlier (3, 73–75).

In further efforts to include as many relevant studies as were available, we chose not to exclude the results from the two studies that examined older adults exclusively (45, 48). All other retrieved studies limited participants to those of adult ages yet pioneering studies of glucocorticoid resistance in the depressed elderly provided early evidence that standard measures of glucocorticoid resistance are valid in a population of advanced age, even in the context of elevated levels of dementia and chronic medical conditions (76, 77). Indeed, glucocorticoid resistance measured using the DST has been validated by meta-analysis to be able to distinguish severely depressed, psychotic individuals from those with more mild disease (78); elderly depressed patients are more likely to experience psychotic symptoms than their younger counterparts. We believe that inclusion of studies that examined both elderly patients and those with medical co-morbidities served to more faithfully model real-world facets of depression and allowed us to include potential results that may represent medical illnesses priming the HPA axis for increased dysfunction in the context of concurrent depression.

Our conclusions are limited by the small number of study subjects included in this review. This directly impacted the precision of effect size estimates, as did the variations in individual study design. Our latter analyses (**Figures 4**–**6**) were particularly at risk of such bias given the need to pool cytokine results. To rule out this possibility, we conducted the same analysis using varied combinations of cytokines from those reported in **Figures 4** and **5**, finding that these ancillary analyses did not differ significantly from the results reported (data not shown). Nevertheless, there is a need for large studies specifically designed to examine the association between glucocorticoid resistance and cytokine production in depression. These studies should include multiple objective measures of glucocorticoid resistance including cortisol levels, the DST, *GR* expression, and possibly, *in vitro* measures of GR function. A comprehensive characterization of glucocorticoid resistance could then be paired with an analysis of at least plasma levels of the major cytokines reviewed in this work (e.g., IL-6 and TNF-α). Inclusion of medication-free patients may be an important aspect to consider in the design of these studies also as levels of both cortisol and cytokines are modulated by antidepressant treatment (13, 79, 80). The work of Cattaneo et al. (56), which is included in this paper, represents an ideal design template from which to draw upon in future work. Here, medication-free depressed patients and controls were recruited, and expression of multiple cytokines as well as the *GR* was taken as part of a larger study examining immune predictors of antidepressant response. Addition of a measurement of plasma cortisol and a DST in each subject would have created a study tailor-made to examine the questions that we have asked in this review.

Two smaller relevant studies have been conducted that may help to fill this gap temporarily. Vedder et al. (81) elegantly assessed the response of the immune system in real-time before and after exposure to LPS. Using concurrent dexamethasone challenge, the authors demonstrated that in depressed patients who were glucocorticoid resistant, increased IL-6 was produced in response to LPS. This contrasted with IL-6 levels produced by patients with lower levels of glucocorticoid resistance and controls. Therefore, glucocorticoid resistance in depressed patients facilitated IL-6 release. Heiser et al. (79) took an *in vitro* approach to examining the same process. They report that immune cells from depressed patients that were resistant to the anti-proliferative effects of glucocorticoids *in vitro* produced more TNF-α in response to a mitogen than controls. Together with our work, these studies suggest that when glucocorticoid resistance is thoroughly quantified, support for its association with the increased production of pro-inflammatory cytokines can be found.

How depressed patients manifest high levels of inflammation in the face of elevated serum glucocorticoids is a psychiatric paradox. Our work demonstrates that we are still in the throes of disentangling this complex relationship. We believe that glucocorticoid resistance in depressed patients may lead the immune system to escape from the normally restraining function(s) of glucocorticoids. The results we present here provide some support for our hypothesis, but also highlight the need for further work. We are excited to be part of this ongoing search.

## Author Contributions

Conceived of study idea: AP, MH, PZ, CP. Conducted initial literature searches: MH, JR. Conducted literature searches, reviewed retrieved papers, and extracted study data: AP, MH. Performed meta-analysis: AP. Interpreted data: AP, CP. Wrote the first draft of the manuscript: AP. Revised further manuscript drafts in response to feedback from co-authors: AP. Provided project oversight and intellectual guidance: CP.

## Funding

AP is the recipient of career development funding from the Province of British Columbia through the Clinician Investigator Program at the University of British Columbia (UBC), the Detweiler Travelling Fellowship from the Royal College of Physicians and Surgeons of Canada, and the Friedman Award for Scholars in Health from UBC. MAH was supported by an Overseas PhD Studentship from King’s College London. CMP is supported by grants from Immunopsychiatry: a Consortium to Test the Opportunity for Immunotherapeutics in Psychiatry (MR/L014815/1) and the Medical Research Council (UK). Additional support has been provided by the National Institute for Health Research Biomedical Research Centre in Mental Health at South London and Maudsley NHS Foundation Trust and King’s College London.

## Conflict of Interest Statement

PZ: research funding from Janssen Pharmaceutical NV/Janssen Pharmaceutical Companies of Johnson & Johnson. CP: research funding from Janssen Pharmaceutical NV/Janssen Pharmaceutical Companies of Johnson & Johnson; speaker’s fees from Lundbeck and consultation fees from Eleusis Benefit Corporation.

The remaining authors declare that the research was conducted in the absence of any commercial or financial relationships that could be construed as a potential conflict of interest.

## References

[1] Glezer I Rivest S Glucocorticoids: protectors of the brain during innate immune responses. Neuroscientist (2004) 10:538–52. 10.1177/1073858404263494 15534039

[2] McEwen BS Biron CA Brunson KW Bulloch K Chambers WH Dhabhar FS The role of adrenocorticoids as modulators of immune function in health and disease: neural, endocrine and immune interactions. Brain Res Rev (1997) 23:79–133. 10.1016/S0165-0173(96)00012-4 9063588

[3] Silverman MN Sternberg EM Glucocorticoid regulation of inflammation and its functional correlates: from HPA axis to glucocorticoid receptor dysfunction. Ann N Y Acad Sci (2012) 1261:55–63. 10.1111/j.1749-6632.2012.06633.x 22823394 PMC3572859

[4] Carroll BJ Curtis GC Mendels J Cerebrospinal fluid and plasma free cortisol concentrations in depression. Psychol Med (1976) 6:235–44. 10.1017/S0033291700013775 1005564

[5] Carroll BJ Martin FI Davies B Resistance to suppression by dexamethasone of plasma 11-O.H.C.S. Br Med J (1968) 3:285–7. 10.1136/bmj.3.5613.285 PMC1986248 4385601

[6] Carroll BJ Curtis GC Neuroendocrine identification of depressed patients. Aust N Z J Psychiatry (1976) 10:13–20. 10.3109/00048677609159480 1065309

[7] Ceccato F Boscaro M Cushing’s Syndrome: screening and diagnosis. High Blood Press Cardiovasc Prev (2016) 23:209–15. 10.1007/s40292-016-0153-4 27160717

[8] Zunszain PA Anacker C Cattaneo A Carvalho LA Pariante CM Glucocorticoids, cytokines and brain abnormalities in depression. Prog Neuropsychopharmacol Biol Psychiatry (2011) 35:722–9. 10.1016/j.pnpbp.2010.04.011 PMC3513408 20406665

[9] Webster JI Sternberg EM Role of the hypothalamic-pituitary-adrenal axis, glucocorticoids and glucocorticoid receptors in toxic sequelae of exposure to bacterial and viral products. J Endocrinol (2004) 181:207–21. 10.1677/joe.0.1810207 15128270

[10] Nadeau S Rivest S Glucocorticoids play a fundamental role in protecting the brain during innate immune response. J Neurosci (2003) 23:5536–44. 10.1523/JNEUROSCI.23-13-05536.2003 PMC6741270 12843254

[11] Pariante CM Why are depressed patients inflamed? A reflection on 20 years of research on depression, glucocorticoid resistance and inflammation. Eur Neuropsychopharmacol (2017) 27:554–9. 10.1016/j.euroneuro.2017.04.001 28479211

[12] Carroll BJ Greden JF Feinberg M Neuroendocrine disturbances and the diagnosis and aetiology of endogenous depression. Lancet (1980) 1:321–2. 10.1016/S0140-6736(80)90826-0 6101783

[13] Carvalho LA Juruena MF Papadopoulos AS Poon L Kerwin R Cleare AJ Clomipramine in vitro reduces glucocorticoid receptor function in healthy subjects but not in patients with major depression. Neuropsychopharmacology (2008) 33:3182–9. 10.1038/npp.2008.44 PMC3513411 18368033

[14] Moher D Liberati A Tetzlaff J Altman DG Preferred reporting items for systematic reviews and meta-analyses: the PRISMA statement. PLoS Med (2009) 6:e1000097. 10.1371/journal.pmed.1000097 19621072 PMC2707599

[15] Wan X Wang W Liu J Tong T Estimating the sample mean and standard deviation from the sample size, median, range and/or interquartile range. BMC Med Res Methodol (2014) 14:135. 10.1186/1471-2288-14-135 25524443 PMC4383202

[16] Kontopantelis E Reeves D Performance of statistical methods for meta-analysis when true study effects are non-normally distributed: a simulation study. Stat Methods Med Res (2012) 21:409–26. 10.1177/0962280210392008 21148194

[17] Leistner C Menke A How to measure glucocorticoid receptor’s sensitivity in patients with stress-related psychiatric disorders. Psychoneuroendocrinology (2018) 91:235–60. 10.1016/j.psyneuen.2018.01.023 29449045

[18] Review Manager (RevMan) [Computer program]. Version 5.3. Copenhagen: The Nordic Cochrane Centre, The Cochrane Collaboration (2014).

[19] Cubała‚ W Landowski J Dziadziuszko M Chrzanowska A Wielgomas B Zinc, C-reactive protein, and cortisol in major depressive disorder: an exploratory analysis. Trace Elem Electroly (2017) 34:104–6. 10.5414/TEX01461

[20] Cubała WJ Landowski J C-reactive protein and cortisol in drug-naïve patients with short-illness-duration first episode major depressive disorder: possible role of cortisol immunomodulatory action at early stage of the disease. J Affect Disord (2014) 152-154:534–7. 10.1016/j.jad.2013.10.004 24161452

[21] Alesci S Martinez PE Kelkar S Ilias I Ronsaville DS Listwak SJ Major depression is associated with significant diurnal elevations in plasma interleukin-6 levels, a shift of its circadian rhythm, and loss of physiological complexity in its secretion: clinical implications. J Clin Endocrinol Metab (2005) 90:2522–30. 10.1210/jc.2004-1667 15705924

[22] Allen AP Naughton M Dowling J Walsh A O’Shea R Shorten G Kynurenine pathway metabolism and the neurobiology of treatment-resistant depression: comparison of multiple ketamine infusions and electroconvulsive therapy. J Psychiatr Res (2018) 100:24–32. 10.1016/j.jpsychires.2018.02.011 29475018

[23] Anisman H Ravindran AV Griffiths J Merali Z Endocrine and cytokine correlates of major depression and dysthymia with typical or atypical features. Mol Psychiatry (1999) 4:182–8. 10.1038/sj.mp.4000436 10208451

[24] Bauer ME Papadopoulos A Poon L Perks P Lightman SL Checkley S Altered glucocorticoid immunoregulation in treatment resistant depression. Psychoneuroendocrinology (2003) 28:49–65. 10.1016/S0306-4530(02)00009-4 12445836

[25] Carvalho LA Torre JP Papadopoulos AS Poon L Juruena MF Markopoulou K Lack of clinical therapeutic benefit of antidepressants is associated overall activation of the inflammatory system. J Affect Disord (2013) 148:136–40. 10.1016/j.jad.2012.10.036 23200297

[26] Darko DF Gillin JC Risch SC Bulloch K Golshan S Tasevska Z Mitogen-stimulated lymphocyte proliferation and pituitary hormones in major depression. Biol Psychiatry (1989) 26:145–55. 10.1016/0006-3223(89)90018-8 2544231

[27] Du Y Li B Zhang H Cao Y Duan X Gong W Airway inflammation and hypothalamic-pituitary-adrenal axis activity in asthmatic adults with depression. J Asthma (2013) 50:274–81. 10.3109/02770903.2013.765891 23323569

[28] Fitzgerald P O’Brien SM Scully P Rijkers K Scott LV Dinan TG Cutaneous glucocorticoid receptor sensitivity and pro-inflammatory cytokine levels in antidepressant-resistant depression. Psychol Med (2006) 36:37–43. 10.1017/S003329170500632X 16255837

[29] Humphreys D Schlesinger L Lopez M Araya AV Interleukin-6 production and deregulation of the hypothalamic-pituitary-adrenal axis in patients with major depressive disorders. Endocrine (2006) 30:371–6. 10.1007/s12020-006-0016-1 17526950

[30] Jozuka H Jozuka E Takeuchi S Nishikaze O Comparison of immunological and endocrinological markers associated with major depression. J Int Med Res (2003) 31:36–41. 10.1177/147323000303100106 12635532

[31] Kaestner F Hettich M Peters M Sibrowski W Hetzel G Ponath G Different activation patterns of proinflammatory cytokines in melancholic and non-melancholic major depression are associated with HPA axis activity. J Affect Disord (2005) 87:305–11. 10.1016/j.jad.2005.03.012 15951024

[32] Kahl KG Herrmann J Stubbs B Krüger THC Cordes J Deuschle M Pericardial adipose tissue and the metabolic syndrome is increased in patients with chronic major depressive disorder compared to acute depression and controls. Prog Neuropsychopharmacol Biol Psychiatry (2017) 72:30–5. 10.1016/j.pnpbp.2016.08.005 27528109

[33] Kahl KG Schweiger U Pars K Kunikowska A Deuschle M Gutberlet M Adrenal gland volume, intra-abdominal and pericardial adipose tissue in major depressive disorder. Psychoneuroendocrinology (2015) 58:1–8. 10.1016/j.psyneuen.2015.04.008 25935636

[34] Kahl KG Bester M Greggersen W Rudolf S Dibbelt L Stoeckelhuber BM Visceral fat deposition and insulin sensitivity in depressed women with and without comorbid borderline personality disorder. Psychosom Med (2005) 67:407–12. 10.1097/01.psy.0000160458.95955.f4 15911903

[35] Karlović D Serretti A Vrkić N Martinac M Marčinko D Serum concentrations of CRP, IL-6, TNF-α and cortisol in major depressive disorder with melancholic or atypical features. Psychiatry Res (2012) 198:74–80. 10.1016/j.psychres.2011.12.007 22386567

[36] Landmann R Schaub B Link S Wacker HR Unaltered monocyte function in patients with major depression before and after three months of antidepressive therapy. Biol Psychiatry (1997) 41:675–81. 10.1016/S0006-3223(96)00176-X 9066991

[37] Lamers F Vogelzangs N Merikangas KR de Jonge P Beekman ATF Penninx BWJH Evidence for a differential role of HPA-axis function, inflammation and metabolic syndrome in melancholic versus atypical depression. Mol Psychiatry (2013) 18:692–9. 10.1038/mp.2012.144 23089630

[38] Lisi L Camardese G Treglia M Tringali G Carrozza C Janiri L Monocytes from depressed patients display an altered pattern of response to endotoxin challenge. PLoS ONE (2013) 8:e52585. 10.1371/journal.pone.0052585 23300980 PMC3536788

[39] Lopes RP Grassi-Oliveira R de Almeida LR Stein LM Luz C Teixeira AL Neuroimmunoendocrine interactions in patients with recurrent major depression, increased early life stress and long-standing posttraumatic stress disorder symptoms. Neuroimmunomodulation (2012) 19:33–42. 10.1159/000327352 22067620

[40] Maes M Bosmans E Meltzer HY Scharpé S Suy E Interleukin-1 beta: a putative mediator of HPA axis hyperactivity in major depression? Am J Psychiatry (1993) 150:1189–93. 10.1176/ajp.150.8.1189 8328562

[41] Maes M Bosmans E Meltzer HY Immunoendocrine aspects of major depression. Eur Arch Psychiatry Clin Neurosci (1995) 245:172–8. 10.1007/BF02193091 7669825

[42] Maes M Scharpé S Meltzer HY Bosmans E Suy E Calabrese J Relationships between interleukin-6 activity, acute phase proteins, and function of the hypothalamic-pituitary-adrenal axis in severe depression. Psychiatry Res (1993) 49:11–27. 10.1016/0165-1781(93)90027-E 7511248

[43] Marques-Deak AH Neto FL Dominguez WV Solis AC Kurcgant D Sato F Cytokine profiles in women with different subtypes of major depressive disorder. J Psychiatr Res (2007) 41:152–9. 10.1016/j.jpsychires.2005.11.003 16375926

[44] Martinac M Babić D Bevanda M Vasilj I Glibo DB Karlović D Activity of the hypothalamic-pituitary-adrenal axis and inflammatory mediators in major depressive disorder with or without metabolic syndrome. Psychiatr Danub (2017) 29:39–50. 10.24869/psyd.2017.39 28291973

[45] Nikkheslat N Zunszain PA Horowitz MA Barbosa IG Parker JA Myint A Insufficient glucocorticoid signaling and elevated inflammation in coronary heart disease patients with comorbid depression. Brain Behav Immun (2015) 48:8–18. 10.1016/j.bbi.2015.02.002 25683698

[46] Rudzki L Pawlak D Pawlak K Waszkiewicz N Małus A Konarzewska B Immune suppression of IgG response against dairy proteins in major depression. BMC Psychiatry (2017) 17:268. 10.1186/s12888-017-1431-y 28738849 PMC5525306

[47] Simmons WK Burrows K Avery JA Kerr KL Taylor A Bodurka J Appetite changes reveal depression subgroups with distinct endocrine, metabolic, and immune states. Mol Psychiatry (2018). 10.1038/s41380-018-0093-6 PMC6292746 29899546

[48] Trzonkowski P Myśliwska J Godlewska B Szmit E Łukaszuk K Wieckiewicz J Immune consequences of the spontaneous pro-inflammatory status in depressed elderly patients. Brain Behav Immun (2004) 18:135–48. 10.1016/S0889-1591(03)00111-9 14759591

[49] Verduijn J Milaneschi Y Schoevers RA van Hemert AM Beekman ATF Penninx BWJH Pathophysiology of major depressive disorder: mechanisms involved in etiology are not associated with clinical progression. Transl Psychiatry (2015) 5:e649. 10.1038/tp.2015.137 26418277 PMC5545638

[50] Weinstein AA Deuster PA Francis JL Bonsall RW Tracy RP Kop WJ Neurohormonal and inflammatory hyper-responsiveness to acute mental stress in depression. Biol Psychol (2010) 84:228–34. 10.1016/j.biopsycho.2010.01.016 PMC2875322 20117167

[51] Musselman DL Miller AH Porter MR Manatunga A Gao F Penna S Higher than normal plasma interleukin-6 concentrations in cancer patients with depression: preliminary findings. Am J Psychiatry (2001) 158:1252–7. 10.1176/appi.ajp.158.8.1252 11481159

[52] Soygur H Palaoglu O Akarsu ES Cankurtaran ES Ozalp E Turhan L Interleukin-6 levels and HPA axis activation in breast cancer patients with major depressive disorder. Prog Neuropsychopharmacol Biol Psychiatry (2007) 31:1242–7. 10.1016/j.pnpbp.2007.05.001 17587477

[53] Marks R Barlow JW Funder JW Steroid-induced vasoconstriction: glucocorticoid antagonist studies. J Clin Endocrinol Metab (1982) 54:1075–7. 10.1210/jcem-54-5-1075 7061698

[54] Carvalho LA Juruena MF Papadopoulos AS Poon L Cleare AJ Pariante CM reply: ‘clomipramine and glucocorticoid receptor function’. Neuropsychopharmacology (2009) 34:2194–5. 10.1038/npp.2009.43 PMC3513411 18368033

[55] Carvalho LA Bergink V Sumaski L Wijkhuijs J Hoogendijk WJ Birkenhager TK Inflammatory activation is associated with a reduced glucocorticoid receptor alpha/beta expression ratio in monocytes of inpatients with melancholic major depressive disorder. Transl Psychiatry (2014) 4:e344. 10.1038/tp.2013.118 24424390 PMC3905228

[56] Cattaneo A Gennarelli M Uher R Breen G Farmer A Aitchison KJ Candidate genes expression profile associated with antidepressants response in the GENDEP study: differentiating between baseline ‘predictors’ and longitudinal ‘targets’. Neuropsychopharmacology (2013) 38:377–85. 10.1038/npp.2012.191 PMC3547188 22990943

[57] Miller AH Pariante CM Pearce BD Effects of cytokines on glucocorticoid receptor expression and function. Adv Exp Med Biol (1999) 461:107–16. 10.1007/978-0-585-37970-8_7 10442170

[58] Morrison MF Redei E TenHave T Parmelee P Boyce AA Sinha PS Dehydroepiandrosterone sulfate and psychiatric measures in a frail, elderly residential care population. Biol Psychiatry (2000) 47:144–50. 10.1016/S0006-3223(99)00099-2 10664831

[59] Oldehinkel AJ van den Berg MD Flentge F Bouhuys AL Horst GJ Ormel J Urinary free cortisol excretion in elderly persons with minor and major depression. Psychiatry Res (2001) 104:39–47. 10.1016/S0165-1781(01)00300-6 11600188

[60] Strickland PL Deakin JFW Percival C Dixon J Gater RA Goldberg DP Bio-social origins of depression in the community. Br J Psychiatry (2002) 180:168–73. 10.1192/bjp.180.2.168 11823330

[61] Fries E Hesse J Hellhammer J Hellhammer DH A new view on hypocortisolism. Psychoneuroendocrinology (2005) 30:1010–6. 10.1016/j.psyneuen.2005.04.006 15950390

[62] Morrison MF Ten Have T Freeman EW Sammel MD Grisso JA DHEA-S levels and depressive symptoms in a cohort of African American and Caucasian women in the late reproductive years. Biol Psychiatry (2001) 50:705–11. 10.1016/S0006-3223(01)01169-6 11704078

[63] Hellhammer J Schlotz W Stone AA Pirke KM Hellhammer D Allostatic load, perceived stress, and health: a prospective study in two age groups. Ann N Y Acad Sci (2004) 1032:8–13. 10.1196/annals.1314.002 15677392

[64] Nilsonne G Lekander M Åkerstedt T Axelsson J Ingre M Diurnal variation of circulating interleukin-6 in humans: a meta-analysis. PLoS ONE (2016) 11:e0165799. 10.1371/journal.pone.0165799 27832117 PMC5104468

[65] Carvalho LA Urbanova L Hamer M Hackett RA Lazzarino AI Steptoe A Blunted glucocorticoid and mineralocorticoid sensitivity to stress in people with diabetes. Psychoneuroendocrinology (2015) 51:209–18. 10.1016/j.psyneuen.2014.09.023 PMC4275581 25462894

[66] Gaudiano BA Epstein-Lubow G Miller IW Does the dexamethasone suppression test reliably discriminate between psychotic and nonpsychotic major depression? An exploratory analysis of potential confounds. J Nerv Ment Dis (2009) 197:395–400. 10.1097/NMD.0b013e3181a775cf 19525738 PMC3676665

[67] Matsubara T Funato H Kobayashi A Nobumoto M Watanabe Y Reduced glucocorticoid receptor alpha expression in mood disorder patients and first-degree relatives. Biol Psychiatry (2006) 59:689–95. 10.1016/j.biopsych.2005.09.026 16458268

[68] Pariante CM Miller AH Glucocorticoid receptors in major depression: relevance to pathophysiology and treatment. Biol Psychiatry (2001) 49:391–404. 10.1016/S0006-3223(00)01088-X 11274650

[69] Miller GE Rohleder N Stetler C Kirschbaum C Clinical depression and regulation of the inflammatory response during acute stress. Psychosom Med (2005) 67:679–87. 10.1097/01.psy.0000174172.82428.ce 16204423

[70] Doolin K Farrell C Tozzi L Harkin A Frodl T O’Keane V Diurnal hypothalamic-pituitary-adrenal axis measures and inflammatory marker correlates in major depressive disorder. Int J Mol Sci (2017) 18(10):E226. 10.3390/ijms18102226 29064428 PMC5666905

[71] Stelzhammer V Haenisch F Chan MK Cooper JD Steiner J Steeb H Proteomic changes in serum of first onset, antidepressant drug-naïve major depression patients. Int J Neuropsychopharmacol (2014) 17:1599–608. 10.1017/S1461145714000819 24901538

[72] Maes M Bosmans E Suy E Vandervorst C DeJonckheere C Raus J Depression-related disturbances in mitogen-induced lymphocyte responses and interleukin-1 beta and soluble interleukin-2 receptor production. Acta Psychiatr Scand (1991) 84:379–86. 10.1111/j.1600-0447.1991.tb03163.x 1746291

[73] Lieber AL Newbury N Use of biologic markers in a general hospital affective disorders program. J Clin Psychiatry (1985) 46:217–21. 3922959

[74] Cooke RG Warsh JJ Stancer HC Hasey GM Jorna T Langlet F Effect of concurrent medical illness on dexamethasone suppression test results in depressed inpatients. Can J Psychiatry (1990) 35:31–5. 10.1177/070674379003500105 2317731

[75] Raskind M Peskind E Rivard MF Veith R Barnes R Dexamethasone suppression test and cortisol circadian rhythm in primary degenerative dementia. Am J Psychiatry (1982) 139:1468–71. 10.1176/ajp.139.11.1468 7137397

[76] Carnes M Smith JC Kalin NH Bauwens SF Effects of chronic medical illness and dementia on the dexamethasone suppression test. J Am Geriatr Soc (1983) 31:269–71. 10.1111/j.1532-5415.1983.tb04869.x 6841854

[77] Tourigny-Rivard MF Raskind M Rivard D The dexamethasone suppression test in an elderly population. Biol Psychiatry (1981) 16:1177–84. 6891606

[78] Nelson JC Davis JM DST studies in psychotic depression: a meta-analysis. Am J Psychiatry (1997) 154:1497–503. 10.1176/ajp.154.11.1497 9356556

[79] Heiser P Lanquillon S Krieg J-Vedder H Differential modulation of cytokine production in major depressive disorder by cortisol and dexamethasone. Eur Neuropsychopharmacol (2008) 18:860–70. 10.1016/j.euroneuro.2008.07.003 18775652

[80] Köhler CA Freitas TH Stubbs B Maes M Solmi M Veronese N Peripheral Alterations in cytokine and chemokine levels after antidepressant drug treatment for major depressive disorder: systematic review and meta-analysis. Mol Neurobiol (2018) 55:4195–206. 10.1007/s12035-017-0632-1 28612257

[81] Vedder H Schreiber W Schuld A Kainz M Lauer CJ Krieg J Immune-endocrine host response to endotoxin in major depression. J Psychiatr Res (2007) 41:280–9. 10.1016/j.jpsychires.2006.07.014 17045296

